# Harnessing interpretable and unsupervised machine learning to address big data from modern X-ray diffraction

**DOI:** 10.1073/pnas.2109665119

**Published:** 2022-06-09

**Authors:** Jordan Venderley, Krishnanand Mallayya, Michael Matty, Matthew Krogstad, Jacob Ruff, Geoff Pleiss, Varsha Kishore, David Mandrus, Daniel Phelan, Lekhanath Poudel, Andrew Gordon Wilson, Kilian Weinberger, Puspa Upreti, Michael Norman, Stephan Rosenkranz, Raymond Osborn, Eun-Ah Kim

**Affiliations:** ^a^Department of Physics, Cornell University, Ithaca, NY 14853;; ^b^Materials Science Division, Argonne National Laboratory, Lemont, IL 60439;; ^c^Cornell High Energy Synchrotron Source, Cornell University, Ithaca, NY 14853;; ^d^Department of Computer Science, Cornell University, Ithaca, NY 14853;; ^e^Department of Materials Science and Engineering, University of Tennessee, Knoxville, TN 37996;; ^f^Department of Materials Science and Engineering, University of Maryland, College Park, MD 20742;; ^g^Center for Neutron Research, National Institute of Standard and Technology, Gaithersburg, MD 20899;; ^h^Courant Institute of Mathematical Sciences, New York University, New York, NY 10012;; ^i^Department of Physics, Northern Illinois University, DeKalb, IL 60115

**Keywords:** machine learning, big data, X-ray scattering

## Abstract

The information content of crystalline materials becomes astronomical when collective electronic behavior and their fluctuations are taken into account. In the past decade, improvements in source brightness and detector technology at modern X-ray facilities have allowed a dramatically increased fraction of this information to be captured. Now, the primary challenge is to understand and discover scientific principles from big datasets when a comprehensive analysis is beyond human reach. We report the development of an unsupervised machine learning approach, X-ray diffraction (XRD) temperature clustering (X-TEC), that can automatically extract charge density wave order parameters and detect intraunit cell ordering and its fluctuations from a series of high-volume X-ray diffraction measurements taken at multiple temperatures. We benchmark X-TEC with diffraction data on a quasi-skutterudite family of materials, (Ca*_x_*Sr${}_{1-x}$)_3_Rh_4_Sn_13_, where a quantum critical point is observed as a function of Ca concentration. We apply X-TEC to XRD data on the pyrochlore metal, Cd_2_Re_2_O_7_, to investigate its two much-debated structural phase transitions and uncover the Goldstone mode accompanying them. We demonstrate how unprecedented atomic-scale knowledge can be gained when human researchers connect the X-TEC results to physical principles. Specifically, we extract from the X-TEC–revealed selection rules that the Cd and Re displacements are approximately equal in amplitude but out of phase. This discovery reveals a previously unknown involvement of $5d^{2}$ Re, supporting the idea of an electronic origin to the structural order. Our approach can radically transform XRD experiments by allowing in operando data analysis and enabling researchers to refine experiments by discovering interesting regions of phase space on the fly.

## Significance

Recent instrumental advances now enable large volumes of X-ray diffraction to be collected with high efficiency at synchrotron sources. This article shows that machine learning can produce an unbiased and comprehensive analysis of such data that uniquely combines both long-range and short-range structural correlations as a function of temperature. In Cd_2_Re_2_O_7_, machine learning characterizes both the critical behavior of the primary order parameter and the Goldstone mode fluctuations that drive symmetry breaking at a lower temperature. The approach results from a synergy between computer scientists and physicists, producing a machine learning strategy that is interpretable within the established framework of physics and adaptable to other “big data” problems in materials science and engineering.

**Fig. 1.**
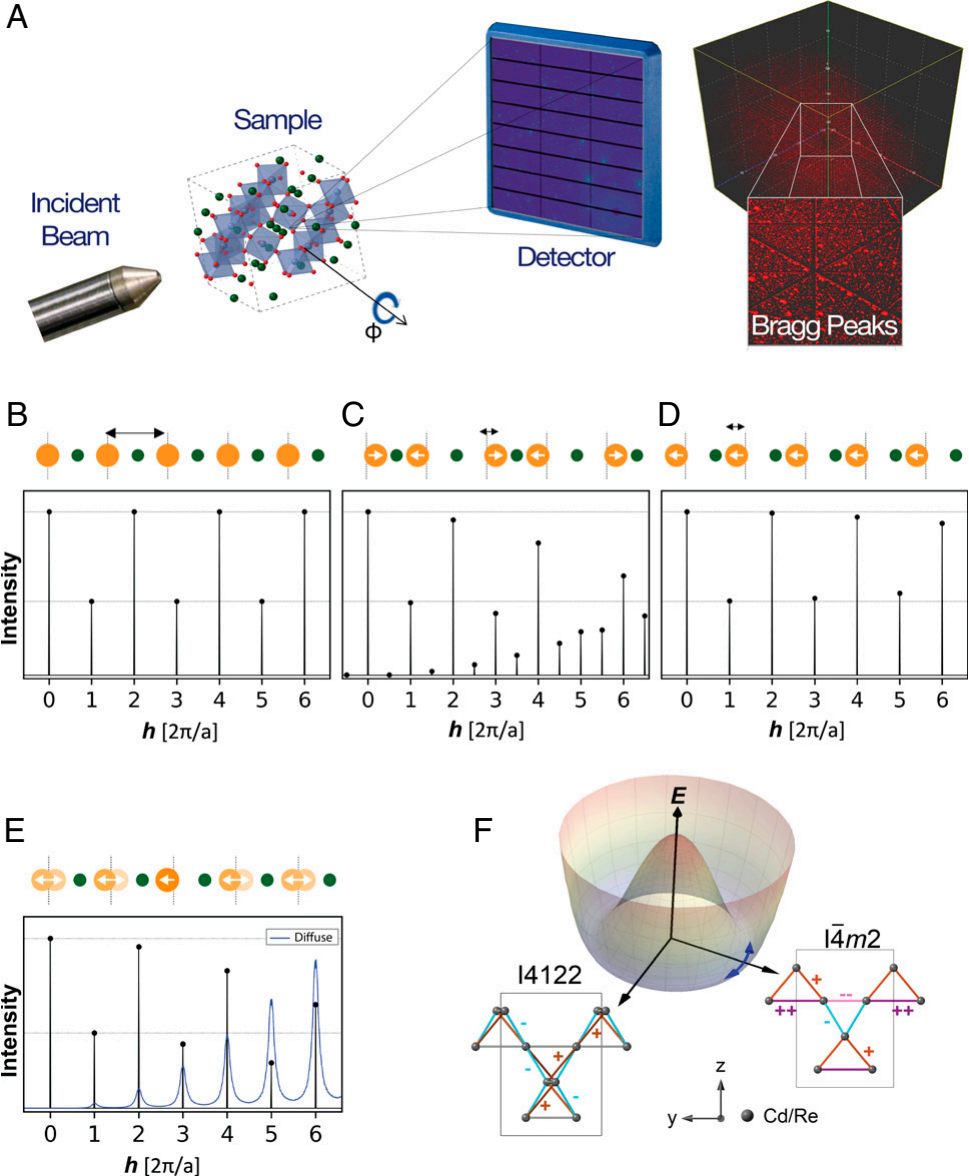
(*A*) Schematic geometry of the X-ray scattering measurements. A monochromatic X-ray beam is incident on the sample, which rotates about the orthogonal ϕ axis while images are captured on a fast area detector. The reciprocal space map shows the $\vec{q}$ coverage of a single plane in the 3D volume after capturing images over a full $360^{{}^{\circ}}$ sample rotation. A 3D volume of reciprocal space covered by the X-ray scattering is shown on the right. Each red dot is a single Bragg peak. With an X-ray energy of 87 keV, a volume of over 10,000 Å^-3^ is measured, containing over 10,000 BZs if the unit cell dimension is 10 Å. Real space positions of atoms (*Top*) and the corresponding scattering intensities (*Bottom*) calculated from simulated 1D crystals with a unit cell containing two atoms, illustrating (*B*) a high-symmetry phase, with (*C*) distortions due to CDW order, (*D*) IUC order, and (*E*) short-range IUC order. In *B*, the high-symmetry phase produces peaks at integer $\vec{q}$. In *C*, displacements of the orange atoms by ±δ double the size of the unit cell producing additional superlattice peaks at half-integer $\vec{q}$ as well as changes in the other peak intensities. See Fig. 2 for X-TEC–aided detection of CDW order in (Ca*_x_*Sr${}_{1-x}$)_3_Rh_4_Sn_13_. In *D*, IUC distortions of the orange atoms by −δ change the peak intensities without producing additional superlattice peaks. In *E*, every orange atom is displaced by ±δ, with a 70% probability of nearest neighbors having the same displacement. This finite correlation length has a small impact on the total scattering (black) but produces broad diffuse scattering (blue, ×70,000 scale compared to total scattering). See Fig. 3 for the detection of IUC distortions and their diffuse scattering in Cd_2_Re_2_O_7_ with X-TEC. (*F*) Bond patterns on the pyrochlore lattice associated with an *E_u_* distortion as inferred in Cd_2_Re_2_O_7_. The two space groups refer to the two different components of *E_u_* with each bond color denoting a different bond length. The amount of distortion of each bond from the average bond (gray) is indicated by ++,−, etc., along with the respective bond color. A Mexican hat potential energy *E* governs the fluctuations between the two *E_u_* components in the broken symmetry phase. See Fig. 4 for the X-TEC–aided resolution of the two *E_u_* components and their fluctuations in Cd_2_Re_2_O_7_.

From the early days of X-ray diffraction (XRD) experiments, they have been used to access atomic-scale information in crystalline materials. The primary challenge has always been how to interpret the angle-dependent scattering intensities of the resultant diffraction patterns (Fig. 1*A*). Bragg and Bragg’s initial insights into how to interpret such data (1) enabled the direct determination of crystal structures for the first time, and they were duly awarded a Nobel prize. Since the phase of the X-ray photon is lost in the measurement, the most common approach to interpreting XRD data is to employ forward modeling using the increasingly sophisticated tools of crystallography developed over the past century. These have been remarkably successful in determining the structure of highly crystalline materials, from simple inorganic solids to complex protein crystals. However, subtle structural changes can be difficult to determine when they only result in marginal changes in intensities without any change in peak locations (2). Furthermore, thermal and quantum fluctuations captured in diffuse scattering away from the Bragg peaks are beyond the reach of conventional crystallographic analysis. The information-rich diffuse scattering is typically weaker than Bragg scattering by several orders of magnitude and can be difficult to differentiate from background noise.

The massive data that modern facilities generate, spanning three-dimensional (3D) reciprocal space volumes that include $O(10^{4})$ Brillouin zones (BZs) (Fig. 1*A*), at rates of $O(10^{2})$ GB/h should capture the systematics of such subtle atomic-scale information. Yet the sheer quantity of data presents a major challenge. Overcoming this challenge is of paramount importance especially in searching for an unknown order parameter and its fluctuations. Specifically, two types of orders and their fluctuations are targets of XRD (see the illustration for a 1D system in Fig. 1 *B*–*E*): those that change the size of the unit cell, such as charge density waves (CDW), and those that involve intraunit cell (IUC) distortions. XRD evidence of CDW order is the emergence of new superlattice peaks, which can be weak and fluctuating, often requiring a targeted search (3, 4). XRD evidence of IUC order is even subtler changes in structure factors of Bragg peaks (5), unless there are changes in extinction rules. However, the ubiquity of electronic nematic order (6, 7) has turned the study of electronically driven IUC order into an increasingly important scientific objective. Electronically driven IUC order and related hidden order phases typically have profound consequences for the electronic structure as revealed by various probes, yet are often accompanied by subtle structural distortions. Examples range from 3d oxides like cuprates, to 4d and 5d oxides like ruthenates and iridates, to 4f and 5f heavy fermion materials like URu_2_Si_2_. These small distortions can challenge conventional crystallographic structural refinement that only tracks Bragg peaks and deduce the structural symmetry by fitting all the atomic positions in a forward model.

As an example of proposed CDW order, the quasi-skutterudite family, (Ca*_x_*Sr${}_{1-x}$)_3_X_4_Sn_13_, where X is a transition metal ion like Co, Rh, or Ir, exhibits marginal Fermi liquid behavior. Much like in cuprates and heavy fermion materials such as YbRh_2_Si_2_, this order can be suppressed to very low temperatures, leading to a linear in temperature resistivity over a large range in temperature. As an example of IUC distortion, in the pyrochlore, Cd_2_Re_2_O_7_, a very subtle structural distortion is associated with large changes in the specific heat and susceptibility. This led Fu (8) to propose the presence of spin nematic order, and some evidence for this was provided by subsequent nonlinear optics measurements (9). Moreover, the inversion breaking structural order itself is novel, whose candidate description by an *E_u_* tensor could support pseudo-Goldstone fluctuations between its two components, $I4_{1}22$ and $I\bar{4}m2$ (Fig. 1*F*) (10). Interestingly, both of these examples exhibit superconductivity at low temperatures, leading to the question of how superconductivity is related to these orders.

To extract atomic-scale information encoded in massive XRD data volumes, much needed is a versatile, interpretable, and scalable approach that can reveal order parameters and fluctuations associated with CDW orders and IUC orders: the vision behind XRD temperature clustering (X-TEC). For the analysis of complex experimental data, dimension reduction and machine learning techniques are increasingly employed (11–18), with an emphasis on supervised learning using hypothesis-driven synthetic data (11–13). To date, most applications of unsupervised techniques to materials data have been limited to exploration of compositional phase diagrams of alloys (19–21). However, an interpretable and unsupervised approach aiming at discovering interaction-driven emergent phenomena in quantum materials such as order parameters and fluctuations can greatly benefit scientific progress.

For versatility, we opted for an unsupervised approach guided by a fundamental principle of statistical mechanics: the balance between the energy (*E*) and entropy (*S*) resting on the temperature (*T*). A change in the collective state of a system occurs in the direction of minimizing the Helmholtz free energy *F* (22):

[1] F=E−TS.

When the temperature *T* is lowered below a certain threshold, the entropy *S* gives way to the ordered state dominated by the system Hamiltonian. Hence, the temperature (*T*) evolution of the XRD intensity for reciprocal space point $\vec{q},\;I_{\vec{q}}(T)$, must be qualitatively different if the given reciprocal space point $\vec{q}$ reflects order parameters or their fluctuations. Tracking the temperature evolution of thousands of BZs to identify systematic trends and correlations in any comprehensive manner is impossible to achieve manually without selection bias. X-TEC embodies the principle of Eq. 1 by clustering the temperature series associated with a given $\vec{q},\;I_{\vec{q}}(T)$, according to qualitative features in the temperature dependence, as in high-dimensional clustering approaches that learn qualitative differences in the voice trains for speaker verification (23). X-TEC achieves interpretability and scalability by using a simple Gaussian mixture model (GMM) (24) at its core and incorporates correlation among nearby $\vec{q}$ points and within and across BZs using label smoothing similar to how signals from different cameras can be correlated for computer vision (25).

## Implementation of X-TEC

**Fig. 2.**
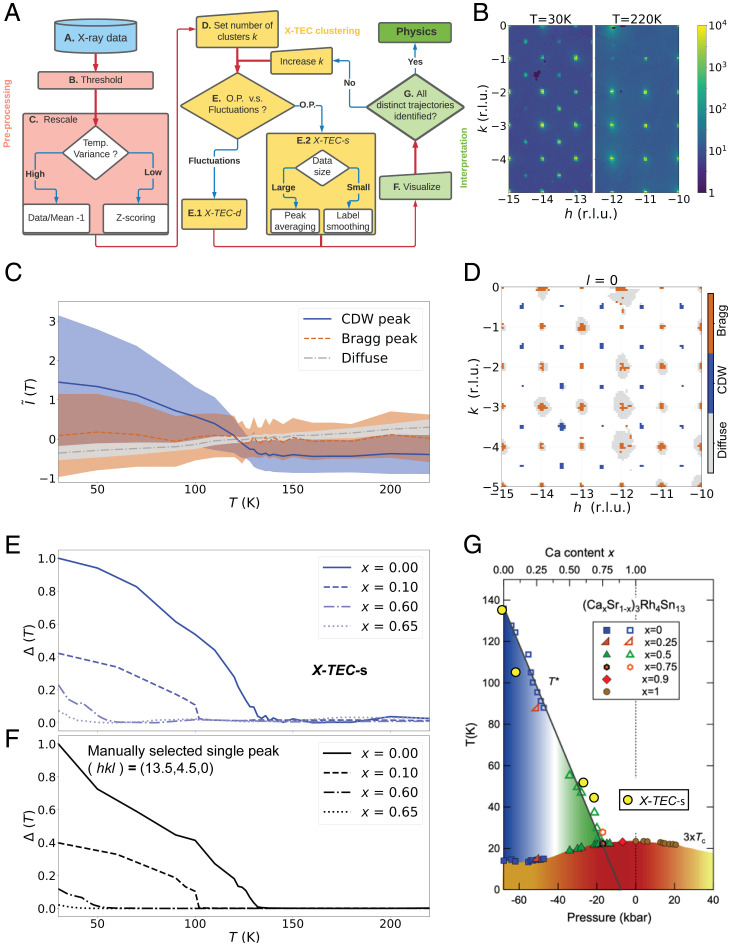
Illustration and benchmarking of X-TEC. (*A*) A flowchart describing the execution of X-TEC. The steps are described in the *Implementation of X-TEC* section and further detailed in *SI Appendix*, section 1. (*B*) Raw XRD image showing a slice of the reciprocal space in the (h,k,l=0) plane, at *T* = 30 K (*Left*) and *T* = 220 K (*Right*). The CDW superlattice peaks are visible at *T* = 30 K and are absent at *T* = 220 K. (*C* and *D*) X-TECs results of the Sr_3_Rh_4_Sn_13_ XRD data with ${\vec{q}}_{i}$ spanning the reciprocal space (h,k,l=0) where h,k∈[−15,15] reciprocal lattice units (r.l.u.). The clustering assignments are color-coded as blue, brown, and gray. In *C*, the lines represent cluster means, and the shaded region shows 1 SD, interpolated between 24 temperature points of measurement. *D* shows the pixels at ${\vec{q}}_{i}$ in the (h,k,0) plane that passed the thresholding (*SI Appendix*, section 1*B*), colored according to their cluster assignments. X-TEC correctly identifies the blue clusters with the CDW super lattice peaks, brown clusters with Bragg peaks, and gray clusters with diffuse scattering. The blue cluster mean (solid line) in *C* represents the rescaled intensity trajectories of all CDW peaks in the data. (*E*) An order parameter like quantity Δ(T) is estimated from the CDW (blue) cluster and is shown for four samples at different values of Ca doping *x*. The Δ(T) is estimated from the cluster means by subtracting the minimum from each cluster mean and appropriate normalization. (*F*) Δ(T) extracted from a manually selected CDW peak at (h,k,l)=(13.5,4.5,0) for the four Ca doping *x* shows a qualitatively similar trajectory to that of X-TEC in *E*. (*G*) The critical temperatures estimated from the X-TEC extracted Δ(T) (yellow filled circles) overlaid onto the known phase diagram from ref. 26 based on phase boundaries from thermodynamic measurements and transport.

In Fig. 2*A*, we provide a flowchart giving a bird’s-eye view of the X-TEC execution. We briefly describe the steps below and provide further details in *SI Appendix*, section 1. Comprehensive XRD temperature series data are obtained for each point $\left\{\vec{q}=(q_{x},q_{y},q_{z})\right\}$ spanning $∼10^{9}$ grid points in a 3D reciprocal space, at 10 to 30 temperatures (step A). The raw data are first passed through a thresholding algorithm that identifies and removes the overwhelming low-intensity background (step B). Next, the intensities, $\left\{I_{{\vec{q}}_{i}}(T_{j}),\;j=1,\cdots ,d^{T}\right\}$ at points $\left\{{\vec{q}}_{i}\right\}$ that passed the thresholding, undergo a rescaling to reduce the dynamic range of the intensity scale (step C). At this point, the user has to decide between two modes of rescaling depending on the nature of the data of interest. To focus on intensities that show a large variation in temperature, the user selects a mean based rescaling: ${\tilde{I}}_{{\vec{q}}_{i}}(T_{j})=(I_{{\vec{q}}_{i}}(T_{j})-\mu_{{\vec{q}}_{i}})/\mu_{{\vec{q}}_{i}}$, where $\mu_{{\vec{q}}_{i}}$ is the mean value of the temperature trajectory at ${\vec{q}}_{i}$. On the other hand, if the focus is on subtle changes in the intensity–temperature trajectories (low-variance trajectories), one selects a variance-based rescaling (z-scoring) given by ${\tilde{I}}_{{\vec{q}}_{i}}(T_{j})=(I_{{\vec{q}}_{i}}(T_{j})-\mu_{{\vec{q}}_{i}})/\sigma_{{\vec{q}}_{i}}$, where $\sigma_{{\vec{q}}_{i}}$ is the SD of the temperature trajectory at ${\vec{q}}_{i}$. The preprocessed data $\left\{{\tilde{I}}_{{\vec{q}}_{i}}\}\equiv \{{\tilde{I}}_{{\vec{q}}_{i}}(T_{j});\;j=1,\cdots ,d^{T}\right\}$ are now ready for the X-TEC clustering. At this point, the user sets the number of clusters *K*, starting with an initial guess (step D).

There are two modes for X-TEC clustering: X-TEC smoothed (X-TEC-s) and X-TEC detailed (X-TEC-d). X-TEC-d assigns cluster labels independently to the trajectories at ${\vec{q}}_{i}$, while X-TEC-s incorporates label smoothing among neighboring *q_i_* points within and across BZs. X-TEC-s is best suited for detecting order parameters reflected in the peak centers, while X-TEC-d can probe finer details in the diffuse scattering and reveal the nature of fluctuations in high-resolution data. The user makes a decision (step E) to choose X-TEC-s for order parameters or X-TEC-d for their fluctuations. Using X-TEC-s and X-TEC-d in tandem can reveal systematic correlations between order parameters captured by peak centers and fluctuations captured by diffuse scattering in an unprecedented manner. For X-TEC-s (step E.2), the user can choose the label smoothing approach to enforce local correlations in the cluster label assignments of neighboring ${\vec{q}}_{i}$. If the size of the dataset is large, the user can opt for a faster and rudimentary version of label smoothing enforced through peak averaging, where intensities of connected pixels in reciprocal space are replaced by their pixel-averaged intensity.

Following the X-TEC clustering, the results are visualized and interpreted (step F). The user observes the *K* distinct temperature trajectories of the clustered data as well as the cluster labels assigned to the ${\vec{q}}_{i}$ points in reciprocal space. The visual interpretation aids the user to arrive at the optimal number of clusters *K* such that increasing *K* does not reveal any more distinct trajectories (step G). The clustered trajectories and their labels in $\vec{q}$ space are now ready for interpretation to aid possible new discoveries such as the identification of hidden orders and selection rules.

At the heart of X-TEC-d is the standard GMM applied to the temperature series, $\left\{{\tilde{I}}_{{\vec{q}}_{i}}\right\}$, treated as a point in the *d^T^*-dimensional space. With the number of clusters *K*, X-TEC-d attempts to model each point in the dataset $\left\{{\tilde{I}}_{{\vec{q}}_{i}}\right\}$ to be independently and identically drawn from a weighted sum of *K* distinct multivariate normal distributions. The hyperparameters to be learned are the mixing weights *π_k_*, *d^T^*-dimensional means $m_{k}$, and $d^{T}\times d^{T}$-dimensional covariances $s_{k},\;(\pi ,m,s)\equiv \{(\pi_{k},m_{k},s_{k});k=1,\cdots ,K\}$. The associated model log-likelihood is

[2] $$\log\;p(\{{\tilde{I}}_{{\vec{q}}_{i}}\}|\pi ,m,s)=\sum_{{\vec{q}}_{i}}\log\;\left[\sum_{k=1}^{K}\pi_{k}N({\tilde{I}}_{{\vec{q}}_{i}}|m_{k},s_{k})\right]\text{​}.$$

Here $N({\tilde{I}}_{{\vec{q}}_{i}}|m_{k},s_{k})$ is the probability density for the *k*th multivariate Gaussian with mean $m_{k}$ and covariance $s_{k}$ evaluated at ${\tilde{I}}_{{\vec{q}}_{i}}$, i.e.,

[3] $$\begin{array}{ll} & N({\tilde{I}}_{{\vec{q}}_{i}}|m_{k},s_{k})\\ & \;\;\equiv \frac{1}{{\left(2\pi \right)}^{d_{T}/2}}\frac{1}{\sqrt{\det\;s_{k}}}e^{-\frac{1}{2}[{\left({\tilde{I}}_{{\vec{q}}_{i}}-m_{k}\right)}^{†}s_{k}^{-1}({\tilde{I}}_{{\vec{q}}_{i}}-m_{k})]}. \end{array}$$

The probability, $w_{i}^{k}$, that the temperature series labeled by ${\vec{q}}_{i}$ belongs to the *k*th cluster is

[4] $$w_{i}^{k}=\frac{\pi_{k}N({\tilde{I}}_{{\vec{q}}_{i}}|m_{k},s_{k})}{\sum_{k}\pi_{k}N({\tilde{I}}_{{\vec{q}}_{i}}|m_{k},s_{k})},$$

according to Bayes’ theorem. X-TEC learns the hyperparameters (π,m,s) using a stepwise expectation maximization (EM) algorithm (27) (*SI Appendix*, section 1*H*). Much like mean-field theory familiar to physicists, the EM algorithm iteratively searches for the saddle point of the lower bound of the log-likelihood

[5] $$\begin{array}{ll} \tilde{\ell }(\{w_{i}^{k},\pi_{k},m_{k},s_{k}\})= & \sum_{i,k}w_{i}^{k}\log\;[\frac{\pi_{k}N({\tilde{I}}_{{\vec{q}}_{i}}|m_{k},s_{k})}{w_{i}^{k}}]\\ & +\lambda (1-\sum_{k}\pi_{k}), \end{array}$$

where *λ* is a Lagrange multiplier. The cluster assignment of a given reciprocal space point ${\vec{q}}_{i}$ is then determined by the converged value of the clustering expectation ${\text{arg}\;\text{max}}_{k}\{w_{i}^{k}\}$.

For X-TEC-s with label smoothing, the algorithm first constructs a nearest neighbor graph in momentum space, connecting reciprocal space points that share similar momenta. For each point, the neighbors are weighted by their distance in momentum space and the weights normalized. Label smoothing averages the cluster assignments of a point with its (weighted) neighbors. We incorporate this smoothing step between the E and M step of the GMM.

## CDW Order and X-TEC Benchmarking

In order to demonstrate the power of X-TEC in action and benchmark its results, we first analyze a collection of data in the vicinity of a putative CDW quantum critical point. Sr_3_Rh_4_Sn_13_ is a quasi-skutterudite compound that has a CDW transition at ∼138 K and a superconducting transition at 4.7 K (26). Doping with calcium applies chemical pressure that suppresses the CDW transition, and electrical resistivity and heat capacity experiments on (Ca*_x_*Sr${}_{1-x}$)_3_Rh_4_Sn_13_ provided evidence of a quantum critical point at a composition of *x* = 0.9 (Fig. 2*G*), corresponding to a peak in the superconducting dome (26), reminiscent of the cuprate phase diagram (28). This interpretation was supported both by inelastic X-ray measurements of soft phonon modes (29) and, more recently, X-ray measurements of the CDW order parameter in the related family, (Ca*_x_*Sr${}_{1-x}$)_3_Ir_4_Sn_13_ (30). We have been developing highly efficient methods of mapping out such phase diagrams using high-energy X-rays on Sector 6-ID-D at the Advanced Photon Source using a monochromatic X-ray energy of 87 keV (31). Images are collected on a fast area detector (Pilatus 2M CdTe) at a frame rate of 10 Hz while the sample is continuously rotated through 360${}^{{}^{\circ}}$ at a speed of 1${}^{{}^{\circ}}$/s (Fig. 1*A*). These rotation scans are repeated twice to fill in gaps between the detector chips, so a single measurement represents an uncompressed data volume of over 100 GB collected in under 20 min. This allows comprehensive measurements of the temperature dependence of a material in 12 h or less. Using a cryostream, we are able to vary the temperature from 30 to 300 K. The rotation scans sweep through a large volume of reciprocal space, containing over 10,000 BZs (Fig. 1*A*); when the data are transformed into reciprocal space coordinates, the 3D arrays are typically reduced in size by an order of magnitude. More details of both the measurement and data reduction workflow are given in ref. 31; see also *SI Appendix*, section 1*A* and ref. 32. Fig. 2*B* shows the raw XRD images in the (h,k,l=0) plane, at *T* = 30 and *T* = 220 K. At *T* = 30 K, the CDW superlattice peaks are clearly seen at $q_{\mathrm{CDW}}=(0.5,0.5,0)$ and symmetry equivalents with respect to the cubic Bragg peaks, which are absent at the higher temperature.

In (Ca*_x_*Sr${}_{1-x}$)_3_Rh_4_Sn_13_, we applied X-TEC to the XRD data on four compounds, (x=0,0.1,0.6,0.65), to map out the phase diagram as a function of both temperature and doping automatically. In Fig. 2*C*, we present cluster means and variances of the three-cluster (*K* = 3) results for undoped Sr_3_Rh_4_Sn_13_. The optimal number of clusters is obtained as the minimum number needed to separate the distinct temperature trajectories (*SI Appendix*, section 1*F*). The temperature dependence of the learned means of the blue, brown, and gray clusters makes it evident that the blue cluster represents the order parameter and the temperature at which it falls to 0 is the critical temperature, $T_{c}∼135$ K. The clustering results can be interpreted by locating the cluster assignments in reciprocal space, as shown in Fig. 2*D*. The location of the blue pixels (which correspond to the blue cluster) identifies the ordering wave vector *q_CDW_* as expected from the raw images in Fig. 2*B*. The diffuse scattering is captured by the gray clusters, while the Bragg peaks are captured by the brown clusters. The three clusters are first identified from an X-TEC-d clustering (see *SI Appendix*, section 1*E*, for the X-TEC-d results), and the label smoothing is applied to the blue and brown clusters (peak centers) after excluding the gray diffuse scattering. Label smoothing keeps the clustering output to be smoothly connected in the vicinity of each peak, simplifying interpretation. Plotting the CDW order parameters extracted automatically by X-TEC at each doping, we can track the evolution of the critical temperature *T_c_* as a function of chemical pressure (Fig. 2*G*), allowing us to map out the quantum phase diagram associated with the CDW ordering in (Ca*_x_*Sr${}_{1-x}$)_3_Rh_4_Sn_13_, in a similar way to ref. 30, without any prior knowledge of the wave vectors or transition temperatures.

A comparison of the X-TEC extracted CDW order parameter Δ(T) (Fig. 2*E*) with that from a manually selected superlattice peak (Fig. 2*F*) shows excellent agreement. In the past, we would have analyzed such data by manually identifying a few superlattice peaks, with the assumption that they are representative of the whole, and fitting their temperature dependence. This may be justified in many cases, but in doing so, we would be ignoring over 99% of the data, limiting the statistical precision available from such comprehensive datasets and potentially missing secondary components of the order parameter. X-TEC eliminates the danger of selection bias in such analyses. The large data volume also allows us to utilize the 3D-Δ PDF method (31), in order to determine the nature of the atomic distortions both below and above T*_c_*, which will be discussed in a future publication.

## IUC Order, Fluctuations, and Selection Rules

**Fig. 3.**
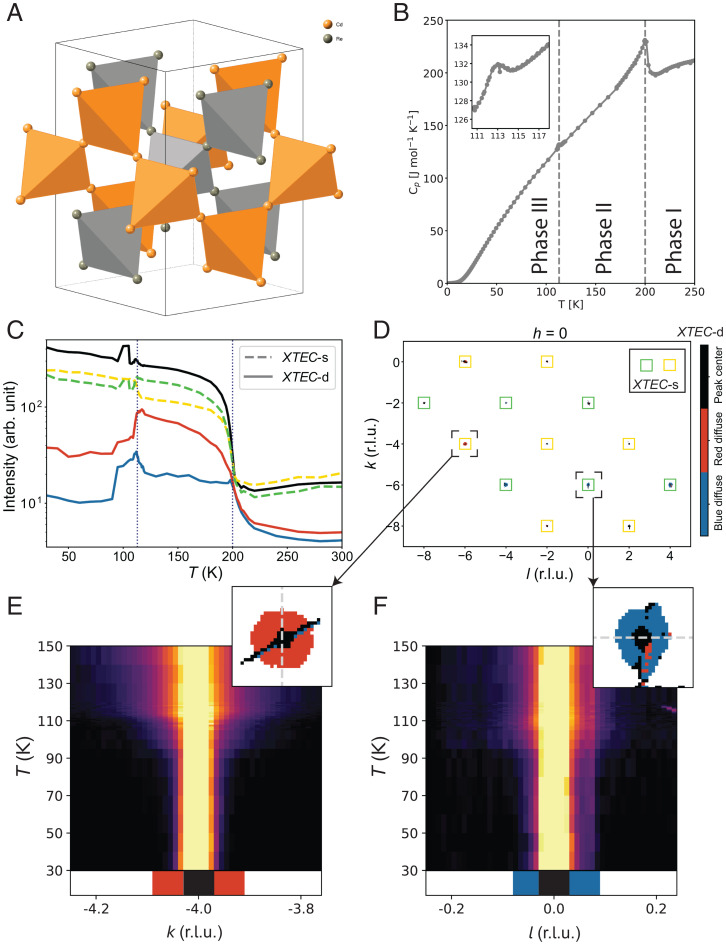
X-TEC analysis of Cd_2_Re_2_O_7_ XRD data. (*A*) Crystal structure of Cd_2_Re_2_O_7_ showing only Cd and Re, in the high-temperature cubic phase. (*B*) Temperature dependence of the specific heat of Cd_2_Re_2_O_7_, showing the second-order phase transition at $T_{s1}$ = 200 K and the first-order phase transition at $T_{s2}$ = 113 K (*SI Appendix*, section 3*A*). Three temperature ranges are marked as phase I ($T>T_{s1}=200\;\text{K}$), phase II ($T_{s2}=113\;\text{K}<T<T_{s1}$), and phase III ($T<T_{s2}$). (*C*) X-TEC results on the cubic forbidden Bragg peaks from high-resolution XRD data, showing temperature dependence of the mean intensity of each cluster (the cluster assignments are obtained from 30 K ≤T≤ 150 K data; see *SI Appendix*, section 3*C*, for details). The lines are average intensity trajectories of their respective cluster assignments from all cubic forbidden Bragg peaks in the data. The solid lines show three-cluster (*K* = 3) X-TEC-d trajectories, color coded as black, red, and blue. The dashed line shows two-cluster (*K* = 2) X-TEC-s (peak averaged) trajectories, colored yellow and green. The temperatures of the two structural phase transitions are shown as dotted lines. (*D*) The X-TEC-d cluster color assignments (black, red, and blue) of the thresholded pixels, as well as X-TEC-s cluster assignments of the Bragg peaks (marked as yellow and green squares centering the Bragg peaks), in a section of the *h* = 0 plane, where *k* and *l* are in r.l.u. The color coding of the clusters is the same as in *C*. (*E* and *F*) The regions in the vicinity of two Bragg peaks at $0\bar{46}$ (*Left*) and $0\bar{6}0$ (*Right*) are magnified to show that the peak centers in both belong to the black cluster, while halos form two distinct clusters (red and blue, respectively) separated from their peak centers. X-TEC-d and X-TEC-s together show that red (blue) diffuse halos and the yellow (green) Bragg peaks lock into a strict one-to-one correspondence with both exhibiting a rigid selection rule. The raw intensity plotted for $0\bar{46}$ (*Left*) and $0\bar{6}0$ (*Right*) along a line cut (the gray dashed line shown in the respective zoom-ins) confirm the temperature dependence of the red and blue halo intensities represented by the cluster means in *C*. Specifically, the $0\bar{46}$ peak has enhanced diffuse scattering above $T_{s2}\approx 113$ K, consistent with the temperature dependence of the red cluster mean. The $0\bar{6}0$ peak shows an anomaly near $T_{s2}$ and a suppressed diffuse scattering above, consistent with the temperature dependence of the blue cluster mean.

We now employ X-TEC-s and X-TEC-d in tandem to study hidden IUC order and order parameter fluctuations in the pyrochlore metal Cd_2_Re_2_O_7_ (33–35) (Fig. 3*A*), whose low-temperature phases have recently attracted much interest and controversies (9, 36–41).

The Cd_2_Re_2_O_7_ goes through a second-order transition at $T_{s1}=200$ K from the cubic pyrochlore $Fd\bar{3}m$ structure (phase I) to a structure that breaks inversion symmetry (phase II), with a large thermodynamic signature in the specific heat (Fig. 3*B*). Most studies conclude that the space group of phase II is the $I\bar{4}m2$ component of *E_u_* symmetry (37). At a lower temperature, a first-order transition at $T_{s2}=113$ K (phase III) is observed and is proposed to arise from the other component of *E_u_*, which is the $I4_{1}22$ space group (37). An additional transition at 80 K is posited following recent Raman data showing line splittings consistent with a lowering to orthorhombic symmetry (speculated to be an *F*222 space group) (42).

The results for phase II are consistent with the picture where $I\bar{4}m2$ and $I4_{1}22$ are the two components of the *E_u_* order parameter, a rank-2 tensor. The degeneracy between these two states is lifted at sixth order in Landau theory (43), resulting in a pseudo-Goldstone mode encoding fluctuations between the two phases (44, 45) (Fig. 1*F*). Raman scattering (10) shows a strong central peak that appears to be the Goldstone mode, along with a higher-frequency mode which appears to be the Higgs mode [although this has been recently questioned based on pump–probe measurements (41)]. The uniqueness of this situation is that although pseudo-Goldstone modes have been seen in other materials, notably ferroelectrics, they typically exist at much higher frequencies (45). The fact that this is not the case for Cd_2_Re_2_O_7_ indicates that the anisotropy in the Landau free energy is anomalously small. Confirmation of such low-frequency fluctuations has so far remained beyond the reach of XRD.

However, the *E_u_* structural order of phase II is now questioned after the discovery of a purported $T_{2u}$ electronic order from second harmonic generation (SHG) (9). While the SHG data also show the *E_u_* structural order, they reveal the surprising fact that the *E_u_* order does not have the expected temperature dependence of a primary order parameter, unlike the $T_{2u}$ signal, which does (9, 38–40). The proposed $I4_{1}22$ space group of phase III is also controversial in that earlier SHG data (36) did not show the expected rotation of the signal from $I\bar{4}m2$ to $I4_{1}22$ that should accompany such a phase transition. A combination of small atomic displacements with crystallographic twinning (46) has made it challenging to determine the true structure of these low-symmetry states using traditional crystallographic approaches (47, 48). The relationship between the *E_u_* structural order and the proposed $T_{2u}$ hidden order indicated by the SHG data has also remained elusive to XRD probes.

We performed X-ray scattering measurements over a wide temperature range (30 K <T<300 K) on a single crystal of Cd_2_Re_2_O_7_, which our measurements show is untwinned, at least in phase II. This may be due to the small volume (400 × 200 × 50 µ m^3^) required for our synchrotron measurements. We first performed scans using an X-ray energy of 87 keV, which contained scattering spanning nearly 15,000 BZs, in order to search for previously undetected peaks and determine the systematic (*HKL*) dependence of the Bragg peak intensities at each temperature (*SI Appendix*, section 3*B*). To better understand the order parameter fluctuations, we then reduced the energy to 60 keV to improve the $\vec{Q}$ resolution and increased the number of temperatures, particularly near the phase transitions. We comprehensively analyzed the resulting datasets (32) with a combined volume of nearly 8 TB using X-TEC-s and X-TEC-d in a time frame of a few minutes (see *SI Appendix*, section 3*C*, for details on preprocessing and CPU times for X-TEC analysis).

**Fig. 4.**
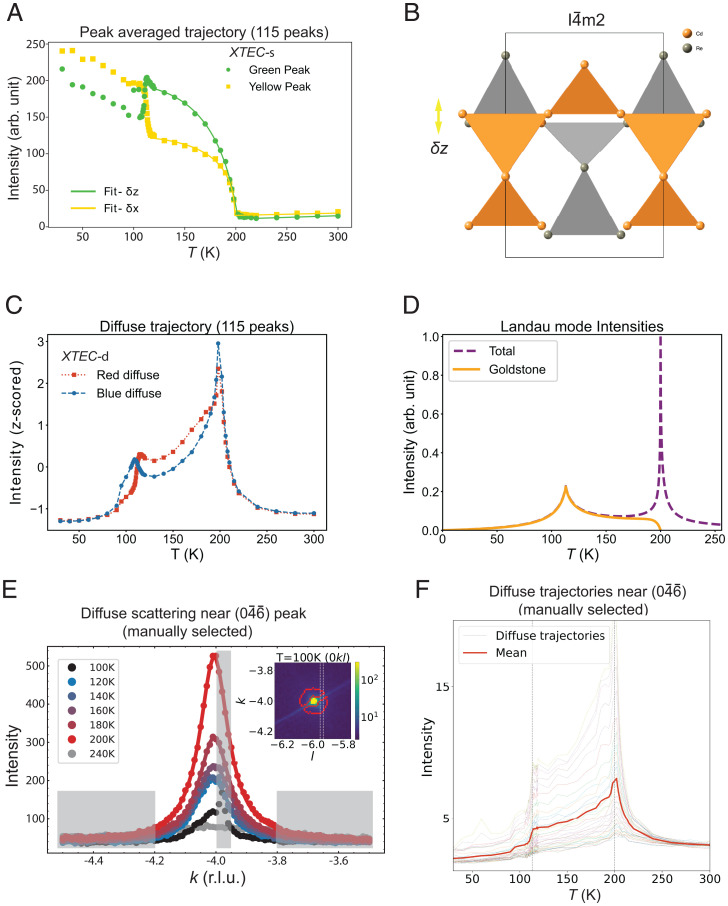
Order parameters and their fluctuations inferred from X-TEC analysis of cubic forbidden Bragg peaks. (*A*) The filled symbols are the two-cluster mean intensity trajectories of peak averaged data (yellow and green trajectories from Fig. 3*C*), and solid lines are fits to these cluster means based on the model assuming *δx* displacements (yellow) and *δz* displacements (green) of cations to vary as ${\left(T-T_{c}\right)}^{\beta }$, with a common order parameter exponent of β=0.25 as discussed in *SI Appendix*, section 3*D*. (*B*) Schematic diagram of the relative *z* axis displacements of cation sublattices for the *Cd* (orange) and *Re* (gray) with respect to the cubic phase, inferred from the fit in *A*. The X-TEC–discovered selection rule and the fit establish the approximately equal magnitude but out-of-phase displacements $\delta z_{Cd}$ and $\delta z_{Re}$. (*C*) The characteristic temperature dependences of the diffuse clusters are revealed by the z-scored intensities (for each intensity, subtract their mean over *T* and then divide their SD in *T*). The red and blue trajectories correspond to the respective cluster average of the z-scored intensities. Lines are guides for the eyes. (*D*) The calculated Landau mode intensities as a function of *T* (*SI Appendix*, section 3*E*). Outside of the critical region near $T_{s1}$ (200 K), the intensity is dominated by the Goldstone mode intensity. Note the resemblance of the calculated intensity to the diffuse trajectory in *C*. (*E*) Main panel shows the temperature dependence of the diffuse scattering line-cut profiles near $\left(0\bar{46}\right)$ Bragg peak, whose intensities are integrated within the manually selected dashed lines in the (0kl) plane, shown in *Inset*. From a visual inspection of their temperature dependence, the shaded gray region is excluded from diffuse scattering. *Inset* shows the intensity distribution in (0kl) plane at 100 K around the $\left(0\bar{46}\right)$ Bragg peak. The red curves enclose the X-TEC-d determined region for diffuse scattering. The red boundary cleverly avoids the diagonal Bragg streak which is not a part of the diffuse scattering (matching the shaded gray region near the peak in the main panel). (*F*) The temperature trajectories of diffuse scattering intensities (dotted lines) and their average intensity (solid line) near the $\left(0\bar{46}\right)$ peak, from the manually selected regions of diffuse scattering in *E*. Vertical dashed lines mark $T_{s1}$ (200 K) and $T_{s2}$ (113 K). The trajectories show the same qualitative features of the X-TEC-d red (square symbol) diffuse trajectory in *C*, with strong scattering at $T_{s1}$ and enhanced intensity above 113 K reflecting the stronger Goldstone fluctuations from *z* axis displacements shown in *B*.

We illustrate the sharp characteristics of the order parameter and its fluctuations by focusing on the cubic-forbidden peaks in Figs. 3 and 4 (see *SI Appendix*, section 3*B*, for the clustering results that selects cubic-forbidden peaks as the order parameter of phase II). Fig. 3*C* shows the *K* = 2 clustering means of X-TEC-s and *K* = 3 clustering means of X-TEC-d on all the cubic-forbidden peaks in the data over the temperature range of [30 K, 150 K].* Both outcomes presented big surprises. First, the X-TEC-s outcome separated the cubic forbidden peaks that behave like the order parameter of phase II into two subgroups: one that quickly flattens in phase II to abruptly rise in phase III (yellow) and the other that continues to rise in phase II to abruptly drop in phase III (green). Second, X-TEC-d clustering separates out the diffuse regions associated with each of the subgroups of cubic-forbidden peaks to define their own clusters with temperature dependencies that are qualitatively different (red and blue in Fig. 3*C*) and distinct from the temperature dependencies of the peak centers.

The reciprocal space distribution of the clusters reveals precise selection rules and tight correlation between the order parameter tracked in X-TEC-s and the fluctuations revealed in X-TEC-d. Due to the orders of magnitude differences in intensity scales, X-TEC-s is dominated by the peak centers. X-TEC-d separated out the peak centers from the halos of diffuse regions. Combining the two results, we present the X-TEC-s outcome through the color of the peak centers detected in X-TEC-d. The (*HKL*) assignments of the two subgroups in X-TEC-s, and their associated diffuse halos in X-TEC-d (Fig. 3*D*), reveal strict selection rules. Yellow peaks (with red halos) are of the form $\left(4n_{1},4n_{2},4n_{3}+2\right)$, while green peaks (with blue halos) have $\left(4n_{1}+2,4n_{2},4n_{3}\right)$ or $\left(4n_{1},4n_{2}+2,4n_{3}\right)$, in the cubic indices of phase I. The mean intensity trajectories of red and blue clusters in Fig. 3*C* indicate that the red halo sustains intensity throughout phase II to only dive down at $T_{s2}=113$ K while the blue halo picks up intensity at around $T_{s2}$ to abruptly die out at around 90 K. The temperature evolution of representative line cuts shown in Fig. 3 *E* and *F* confirm these observations in the raw data.

## Discussion

The systematics in the temperature dependencies of different cubic-forbidden peaks and their diffuse halos revealed using the two modes of X-TEC on the entire 8 TB of data present an unprecedented opportunity to extract atomic-scale clues regarding the hidden order.

First, we can extract an order parameter critical exponent associated with the structural transition that is reflecting the entire dataset from the X-TEC-s mean trajectories. Fig. 4*A* shows the temperature dependence of the two peak averaged clusters (yellow and green) of cubic-forbidden peaks and their fits, in which we treat the displacements as order parameters with a common exponent *β* (*SI Appendix*, section 3*D*). Both clusters fit to the common exponent of β≈0.25 close to $T_{s1}$. This is close to the value expected for a 2D-XY system (49). This is a surprise in that the *E_u_* signal observed by SHG scales linearly in $T_{s1}-T$, which is 4β instead of the expected 2β indicated by theory (38), whereas it is the $T_{2u}$ signal that scales like 2β.

Second, we can convert the selection rule revealed by X-TEC into atomic distortions. The selection rule shows that the two clusters correspond to two distinct classes of structure factor, whose values only depend on the distortions of the Cd and Re sublattices: the yellow cluster consists of peaks that are dominated by *z* axis displacements $\left(\delta z_{\text{Cd}},\delta z_{\text{Re}}\right)$, and those in the green cluster are dominated by in-plane displacements, along *x* or *y* depending on the Wyckoff position, $\left(\delta x_{\text{Cd}},\delta x_{\text{Re}}\right)$ (*SI Appendix*, section 3*D*) (Fig. 4*B*). The flat temperature dependence of the yellow cluster below 180 K results from out-of-phase distortions of the Cd and Re sublattices. The refined values of $(\delta z_{\text{Cd}}$ and $\delta z_{\text{Re}})$ are approximately equal and opposite (Fig. 4*B*). This is another surprising result. Previous refinements (50) indicate that the Re displacements are small, and this is consistent with a density functional theory study (42). Small Re displacements are expected if the 5*d* electrons in Re play a passive role in the structural transition as the Re are in an almost ideally bonded octahedral environment, compared to Cd which is underbonded because of its two short Cd–O and six long Cd–O bonds. Therefore, a large displacement of Re implies that this is a consequence of the $5d^{2}$ configuration of Re being unstable to spin nematic order that should lead to valence bond ordering (different Re–Re bonds, as illustrated in Fig. 1*F*) in a given Re tetrahedron as proposed in other pyrochlores (51).

Third, the connection between the two diffuse halo clusters (red and blue) and the selection rule for the peak centers draws us to the unusual and distinct temperature dependence of the diffuse regions (Fig. 4*C*). Strong critical scattering at $T_{s1}$ is clear in both clusters, but the diffuse contribution is much stronger in the red halo throughout phase II. The role between the two halos reverses at $T_{s2}$. We attribute the fluctuations reflected in the sustained intensity of the red halo to the Goldstone mode manifest through strong *z* axis fluctuations.

To investigate this further, we turn to a description of the various modes (see *SI Appendix*, section 3*E*, for more details of the calculations). Above $T_{s1}$, one has a soft mode whose energy should go to zero at $T_{s1}$. Below this, the soft mode splits into a Higgs mode (fluctuations in the amplitude of the *E_u_* order) and a Goldstone mode (fluctuations in the phase, that is, fluctuations between $I\bar{4}m2$ and $I4_{1}22$). The latter would be at zero energy if there were no anisotropy. In Landau theory, the first anisotropy term appears at sixth order and the next one at eighth order in the free energy. These two must be of opposite sign in order to have a second transition at $T_{s2}$ (43). Their difference changes sign at $T_{s2}$. The net result is that one has a Goldstone mode that starts at zero energy at $T_{s1}$, rises slightly with lowering *T*, then dips down again at $T_{s2}$, and then rises again below this. This can be appreciated by the intensities associated with the various modes (Fig. 4*D*), noting that the Goldstone mode’s coupling to the X-rays is quadratic in the *E_u_* order parameter (52) reflecting the fact that it does not exist above $T_{s1}$ (the analog of the soft mode below $T_{s1}$ is the Higgs mode). From the calculated intensities, one sees that the Goldstone mode completely dominates outside of the critical region near $T_{s1}$. The calculated behavior is remarkably similar to the XRD data (Fig. 4*C*), with a pronounced cusp at $T_{s2}$. This is strong indication that the diffuse scattering is indeed due to structural fluctuations associated with the Goldstone mode.

We now benchmark X-TEC findings of order parameter fluctuations and their coupling to the Bragg peaks against the conventional approach. In the conventional manual approach, one would be forced to select a few Bragg peaks and carefully identify their diffuse region and hope for this hand-picked subset of the data to be representative. The identification of diffuse region in this approach requires tracking temperature dependence of line cuts to separate the diffuse region from the Bragg peak, background scattering, and other streaking artifacts. Fig. 4*E*, Inset, shows that the diffuse region automatically identified by X-TEC is faithful to the conventional definition of the diffuse region. Such a manual approach is laborious at best as apparent from Fig. 4 *E* and *F* and can potentially miss the selection rules governing different Bragg peaks and their diffuse scattering, which are apparent only from an extensive analysis of both the diffuse scattering and the Bragg peaks. We are further limiting the statistical precision available from such comprehensive datasets.

## Summary

In summary, we developed X-TEC, an unsupervised and interpretable ML algorithm for voluminous XRD data that is guided by the fundamental role temperature plays in emergent phenomena. By analyzing the entire dataset over many BZs and making use of temperature evolutions, X-TEC can pick up subtle features representing both order parameters and fluctuations from higher-intensity backgrounds. The two modes, X-TEC-s and X-TEC-d, allow for discovery of systematics in order parameters and its fluctuations despite orders of magnitude differences in intensities. The algorithm is fast with *O*(10) minutes of run time for the tasks presented here. Using X-TEC, we discovered that the superconductor family (Ca*_x_*Sr${}_{1-x}$)_3_Rh_4_Sn_13_ exhibits CDW order, and we mapped out its phase diagram. In Cd_2_Re_2_O_7_, we conclusively identified the primary order parameter of the $T_{s1}=200$ K transition. We further revealed the nature of the IUC atomic distortions in a way that has eluded crystallographic analysis until now. Finally, we revealed XRD evidence of a structural Goldstone mode. The unprecedented degree of microscopic information we have been able to unearth from the XRD is fitting for such comprehensive data but would have been impossible by manual inspection. Instead of determining critical exponents by fitting a handful of peaks, X-TEC provides a means of including the entire data volume by clustering peak intensities from thousands of BZs to produce an analysis that is both robust and rapid in future studies of such phase diagrams. Once X-TEC is integrated to the experimental workflow at the beamline, it can guide the measurements through a real-time analysis of the temperature dependencies. An exciting prospect is to direct the X-TEC extracted data toward automated approaches in inverse scattering problem to efficiently identify the underlying microscopic models (53). Given the general structure of X-TEC, we anticipate it to be broadly applicable to other fields beyond XRD.

## Methods

### Installing X-TEC, Codes, and Tutorials.

The X-TEC codes can be installed through the Python Package Index (PyPI) distribution or from the GitHub source https://github.com/KimGroup/XTEC. The GitHub repository provides instructions to install X-TEC as well as three Jupyter notebook tutorials on X-TEC-d, X-TEC-s with label smoothing, and X-TEC-s with peak averaging.

### The X-TEC Pipeline.

Further details on the X-TEC machinery are provided in *SI Appendix*, section 1, describing the X-ray data collection, the X-TEC processing for the (Ca*_x_*Sr${}_{1-x}$)_3_Rh_4_Sn_13_ data, and the EM algorithm for GMM. *SI Appendix*, section 2, provides another X-TEC benchmarking example with a CDW material: TiSe_2_. The details about the Cd_2_Re_2_O_7_ analysis are provided in *SI Appendix*, section 3.

## Supplementary Material

Supplementary File

## Data Availability

Anonymized HDF5 files, X-TEC codes, and a Jupyter notebook tutorial for X-TEC have been deposited in Analysis of X-rays with Machine Learning and Statistics (AXMAS) Data (DOI: 10.18126/iidy-30e7) (32). Any data not deposited online will be shared with interested researchers upon request.

## Footnotes

^*^For each X-TEC clustering, we increase *K* until there is no gain in information.
