# Supplementary material for: Harnessing interpretable and unsupervised machine learning to address big data from modern X-ray diffraction
Source: Proc Natl Acad Sci U S A. 2022 Jun 9;119(24):e2109665119. doi: 10.1073/pnas.2109665119 (PMC9214512; doi:10.1073/pnas.2109665119)
Supplement: Supplementary File [file pnas.2109665119.sapp.pdf]

Supplementary Information for

Harnessing Interpretable and Unsupervised Machine Learning to Address Big Data from Modern X-ray Diffraction

Jordan Venderley[1], Krishnanand Mallayya[1], Michael Matty[1], Matthew Krogstad[2], Jacob Ruff[3], Geoff Pleiss[4], Varsha Kishore[4], David Mandrus[5], Daniel Phelan[2], Lekhanath Poudel[6,7], Andrew Gordon Wilson[8], Kilian Weinberger[4], Puspa Upreti[2,9], M. R. Norman[2], Stephan Rosenkranz[2], Raymond Osborn[2], Eun-Ah Kim[1*]

[1]Department of Physics, Cornell University
[2]Materials Science Division, Argonne National Laboratory
[3]Cornell High Energy Synchrotron Source, Cornell University
[4]Department of Computer Science, Cornell University
[5]Department of Materials Science and Engineering, University of Tennessee
[6]Department of Materials Science and Engineering, University of Maryland
[7]NIST Center for Neutron Research, National Institute of Standard and Technology
[8]Courant Institute of Mathematical Sciences, New York University
[9]Department of Physics, Northern Illinois University

Eun-Ah Kim
E-mail: eun-ah.kim@cornell.edu

This PDF file includes:

Figs. S1 to S11 (not allowed for Brief Reports)
SI References

Supplementary Information

1. The *X-TEC* Pipeline

In this section, we lay down the details of the various steps indicated in the *X-TEC* flowchart in Fig. 2(a). We provide the example of *X-TEC* analysis of CDW peaks in $Sr_3Rh_4Sn_{13}$ (Fig. 2 of main text) for a demonstration of the various steps in the pipeline. Users can install the *X-TEC* package through the PyPI distribution channel or from the GitHub repository: github.com/KimGroup/XTEC. For a hands-on experience with *X-TEC*, readers are encouraged to explore and modify this analysis with the tutorial notebooks that we share in the GitHub. For a streamlined presentation, we again show the flowchart below where the steps are marked with the corresponding section labels that describe them.

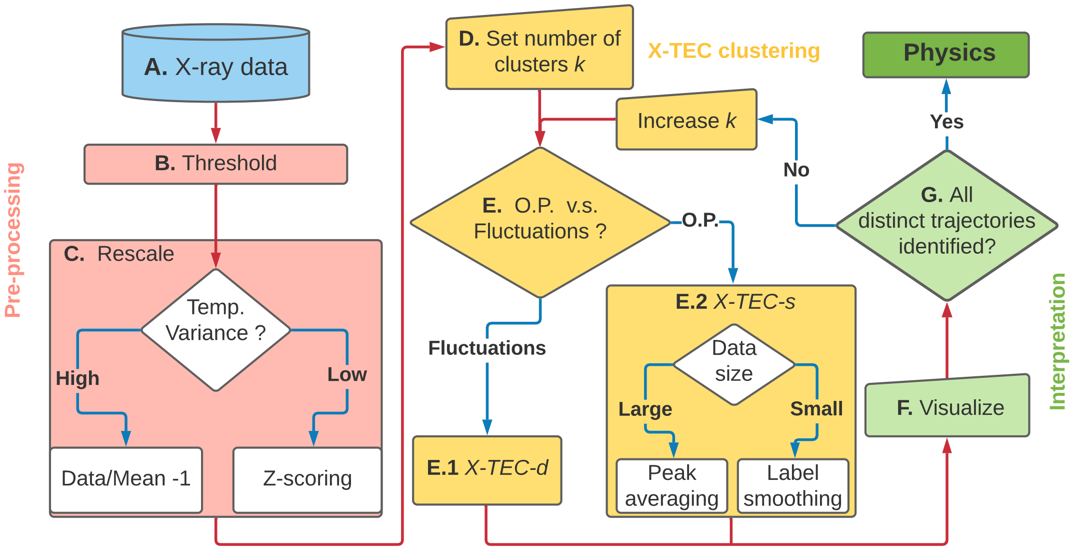

Fig. S1. The flowchart for *X-TEC*. The lettered labels indicate the corresponding subsections in S1 that describe them.

A. X-ray data. A schematic of the x-ray measurement scattering geometry is shown in Fig. 1 of the main article. Three-dimensional volumes of diffuse X-ray scattering were collected at Advanced Photon Source (APS) and CHESS. The APS data were measured on sector 6-ID-D using an incident energy of 87.1 keV and a detector distance of 638 mm, except for the high-resolution measurements on $Cd_2Re_2O_7$, which used an incident energy of 60.0 keV and a distance of 1406 mm. The raw images were collected on a Dectris Pilatus 2M with a 1-mm-thick CdTe sensor layer. The data were collected over a temperature range of 30 K to 300 K, with samples cooled by flowing He gas below 105 K and N_2 gas above 105 K. The CHESS data on $TiSe_2$ were measured on beamline A2 using an incident beam energy of 59 keV and a Dectris Pilatus 6M detector with a 1-mm-thick Si sensor layer. The data were collected over a temperature range of 90 K to 300 K, with samples cooled by flowing N_2 gas. During the measurements, the samples were continuously rotated about an axis perpendicular to the beam at $1°s^{-1}$ over 360°, with images read out every 0.1 s. Three sets of rotation images were collected for each sample at each temperature to fill in gaps between the detector chips. The resulting images were stacked into a three-dimensional array, oriented using an automated peak search algorithm and transformed in reciprocal space coordinates using the software package CCTW (Crystal Coordinate Transformation Workflow), allowing $S(\mathbf{Q})$ to be determined over a range of $\sim \pm 15$ Å^{-1} in all directions ($\sim \pm 6$ Å^{-1} for the high-resolution measurement on $Cd_2Re_2O_7$). Further details are given in Ref. 1. The XRD data for $Sr_3Rh_4Sn_{13}$ can be downloaded from dx.doi.org/10.18126/iidy-30e7

B. Threshold background. A signature difficulty in the analysis of X-ray diffraction data is the existence of physics at several different intensity scales. This is only further exacerbated when probing low-intensity features where the signal-to-noise ratio can be small. If one is to employ thresholding as part of some preprocessing, it is imperative to be careful in order to avoid thresholding-out any important physics. Nevertheless, thresholding is extremely useful for mitigating the influence of noise and for reducing dataset size since most single crystal x-ray diffraction patterns are sparse. Consequently, we propose a new thresholding methodology for isolating the physically relevant regions of k-space.

A naive way to cluster the type of datasets offered by single crystal x-ray diffraction is to apply an i.i.d. assumption and directly try to cluster the associated trajectories, $I_{\vec{q}_i}(T)$, so that each q-point is classified according to its functional temperature dependence. However, such an attempt is immediately thwarted by the existence of a continuum of trajectories spanning over a large intensity range so that getting any meaningful clustering is difficult. The standard way of dealing with this is to use feature scaling a.k.a. standardization in which one removes the mean for each trajectory and then normalizes it by dividing by its standard deviation. However, the dominant features of x-ray diffraction data are usually relatively well-localized peaks

and most trajectories may be attributed to background fluctuations and thermal diffuse scattering. These trajectories have small, finite means and variances so that conventional standardization amplifies the underlying experimental error and noise, thereby spoiling any immediate attempt at clustering. On the other hand, failing to standardize makes it difficult to cluster over different energy scales since low-intensity variations can be washed out by larger ones. Thus some cutoff is needed in order to avoid clustering over noise while maintaining the ability to cluster over different energy scales.

In order to properly threshold our data, we exploit the statistical properties of our trajectories' average intensities, $\log \overline{I_{\vec{q}_i}(T)}$. Here, the average is performed over temperature so that a single average intensity is obtained for each $\vec{q}_i$. Several properties of our data make it advantageous to examine the statistics of $\log \overline{I_{\vec{q}_i}(T)}$ rather than $\overline{I_{\vec{q}_i}(T)}$, most notably its positive semi-definiteness and large range. Since the dominant features our data are naturally sparse and the background trajectories are characterized by possessing small means and variances, we should expect the distribution of $\overline{I_{\vec{q}_i}(T)}$ to be sharply peaked near some relatively small background value. Looking at the logarithm, $\log \overline{I_{\vec{q}_i}(T)}$, broadens this peak allowing us to resolve the finer structural details of this low-intensity background. To first order, we find the distribution of $\log \overline{I_{\vec{q}_i}(T)}$ to be well-characterized by a bulk background contribution that is approximately normally distributed at low intensities with sparsely distributed high intensity contributions. This can be seen in when looking at the distribution of $\log \overline{I_{\vec{q}_i}(T)}$ for the $Sr_3Rh_4Sn_{13}$ data in Fig. S2. In order to separate these high intensity features from rest of the data, we take advantage of their sparsity relative to the background. Specifically, we minimize the Kullback-Leibler divergence D_{KL}, where for probability distributions $p(x), q(x)$:

$$D_{KL}(p(x)||q(x)) = \sum_{x \in X} p(x) \ln \frac{p(x)}{q(x)} \qquad [1]$$

between the distribution of $\log\left(\overline{I_{\vec{q}_i}(T)}\right)$ with a high intensity cutoff and a gaussian. Information theoretically, the Kullback-Leibler divergence quantifies the information loss associated with approximating the distribution $p(x)$ by $q(x)$. In this context, the minimizing D_{KL} optimally chooses a high-intensity cutoff so that the distribution of the remaining $\log \overline{I_{\vec{q}_i}(T)}$ looks closest to a normal distribution. This is illustrated by applying our procedure to the $Sr_3Rh_4Sn_{13}$ data in Fig. S2. Optimization is performed via gradient descent. Note that optimizing with this sliding cutoff is necessary and a Gaussian cannot be directly fitted because the distribution $\log \overline{I_{\vec{q}_i}(T)}$ is heavy tailed. Directly fitting with a Gaussian yields a higher cutoff susceptible to missing important low-intensity features.

The thresholding procedure is the only step in the *X-TEC* pipeline where we analyse the temperature averaged intensity. The rest of the analysis is solely focused on the temperature dependence of these thresholded intensities.

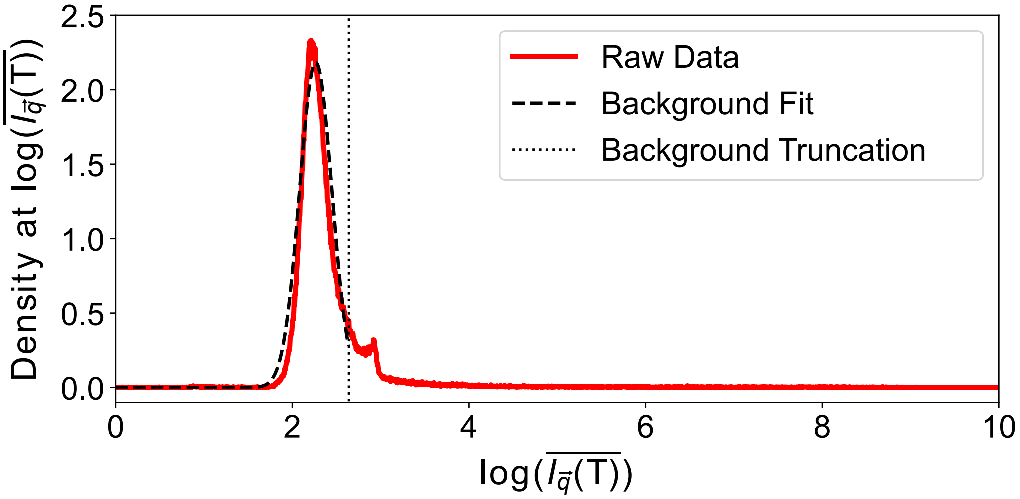

Fig. S2. Histogram (blue) of $\log \overline{I_{\vec{q}}(T)}$ for the $Sr_3Rh_4Sn_{13}$ data, with the background fit (dashed line) and the truncation point (vertical dotted line) determined from a Gaussian fit with a sliding high-intensity cutoff. The algorithm selects the non-Gaussian high intensity features above the cutoff, and these are the intensities that contain the physically relevant signals.

C. Re-scale data. A re-scaling step is necessary to bolster the ability to cluster distinct functional forms of the intensity-temperature trajectories rather than the magnitude of intensities. While there are different ways to re-scale the intensities, we narrow down the choice to two schemes. The user decides the optimal re-scaling procedure depending on the nature of the data and the physics of interest. To focus on trajectories that show high variance in temperature compared to the background (such as the CDW order parameters in $Sr_3Rh_4Sn_{13}$), one re-scales the intensities $\{I_{\vec{q}_i}(T_j);\ j = 1, \ldots, d^T\}$ with their mean at each $\vec{q}_i$ given by,

$$\tilde{I}_{\vec{q}_i}(T_j) = \frac{I_{\vec{q}_i}(T_j)}{\mu_{\vec{q}_i}} - 1, \qquad [2]$$

where $\mu_{\vec{q}_i} = d_T^{-1} \sum_j I_{\vec{q}_i}(T_j)$ is the mean value of the trajectory at $\vec{q}_I$. On the other hand, if the user decides to focus on the low variance trajectories (such as the Goldstone mode fluctuations in $Cd_2Re_2O_7$), a z-scoring is the more efficient choice for rescaling, given by,

$$\tilde{I}_{\vec{q}_i}(T_j) = \frac{I_{\vec{q}_i}(T_j) - \mu_{\vec{q}_i}}{\sigma_{\vec{q}_i}}, \qquad [3]$$

where $\sigma_{\vec{q}_i} = \sqrt{d_T^{-1} \sum_j \left(I_{\vec{q}_i}(T_j) - \mu_{\vec{q}_i}\right)^2}$ is the standard deviation.

D. Select number of clusters K. Following the preprocessing, the user starts with a guess for the number of clusters K. This guess may be physically motivated, such as a prior knowledge on the number of order parameters in the system. The optimal number of clusters for the data is later deduced in step G.

E. *X-TEC*-detailed (*X-TEC*-d) and *X-TEC*-smoothed (*X-TEC*-s). As discussed in the main text, the user decides between the two modalities of $X\text{-}TEC$ depending on whether to focus on order parameter like features from the peak intensities or their fluctuations in the surrounding diffuse scattering.

E.1. X-TEC- detailed. $X\text{-}TEC$-d is the straightforward clustering scheme that clusters the intensity trajectories at different $\vec{q}_i$ independently. This mode is best when provided with high resolution data where resolution limited fluctuations across neighbouring pixels are not significant. As it can probe the trajectory of each pixel independently, it provides the most detailed clustering assignments from the data. This makes it useful for probing distinct behaviours of the diffuse scattering trajectories surrounding the peak centers.

E.2. X-TEC- smoothed. $X\text{-}TEC$-s implements label smoothing as a first order approach for incorporating correlations between nearby momenta and between different Brillouin zones, by allowing labels to diffuse between neighboring points and between unit cells. It ultimately results in cleaner, smoother classifications that better align with physicists intuition for order parameters.

Typical label smoothing is a semi-supervised method in which there exists a ground truth for certain points. These labelings are then “clamped" and diffused through the rest of the system. Here, we lack a bona fide ground truth and so instead incorporate label smoothing dynamically in between the E and M steps of our EM algorithm. Physically, this adds a diffusive “force" to our update scheme that encourages a similar labeling of nearby points and points differing by a reciprocal lattice vector. Convergence in this modified EM method occurs when an equilibrium is reached between this diffusion and the GMM clustering.

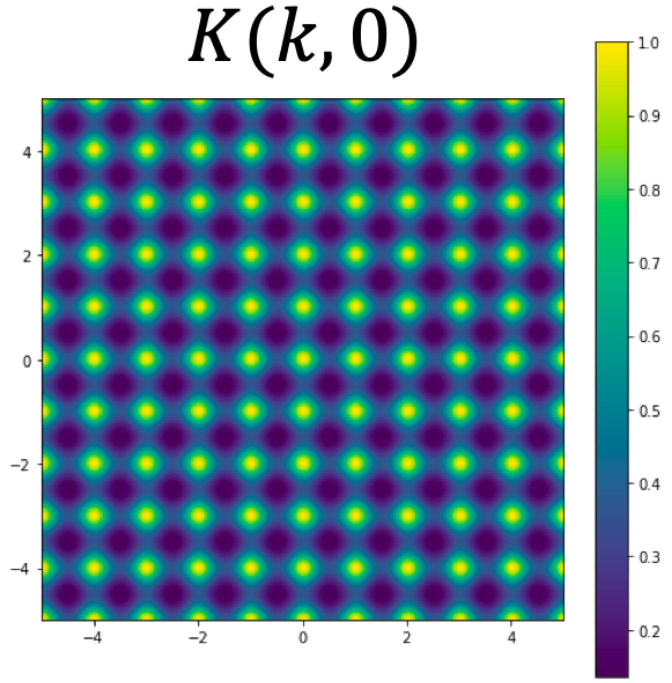

Fig. S3. Kernel, $K(k, 0)$, showing the similarity between the origin and momenta in a 2D.

Our label smoothing requires us to construct a weighted graph connecting similar momenta in order for diffusion to occur. This may be done by computing the following kernel:

$$K(k,k') = \exp\left[-\sum_i \sin^2\left(\frac{Q_i}{2}\cdot(k-k')\right)/\ell^2\right] \qquad [4]$$

where the Q_i are the reciprocal basis vectors and ℓ is the relevant length scale for the local correlations. The structure of this kernel is shown in Fig. S3 where $K(k,0)$ is plotted as an intensity for a 2D slice.

This kernel is really just a weighted adjacency matrix. By incorporating a cutoff in the weights, we may exploit the sparsity of our system for fast matrix-vector multiplication. When handling large datasets, this cutoff is essential since the full kernel is too large to be stored in any reasonable amount of RAM. Define A to be the matrix associated with this kernel after having normalized the rows i.e. it is row stochastic so that $\sum_j A_{ij} = 1$. Now define P to be the matrix consisting of cluster probabilities calculated by the E-step. Specifically, let the first index correspond to the different momenta and the second to the cluster probabilities so that P is also row stochastic. Then the product AP is also row stochastic since $\sum_{jk} A_{ij}P_{jk} = \sum_j A_{ij}(1) = 1$. So by multiplying P by A, we generate a new set of diffused cluster probabilities. The strength of this diffusion can be controlled by the number of matrix multiplications. However, note that we cannot simply apply A until $A^n P$ converges, because the largest eigenvector of A is just the constant vector. In practice, we find that even a single application of A between E- and M-steps is sufficient for obtaining smooth labelings.

When the size of the data is large, one can resort to a cheaper version of label smoothing which is peak averaging. With peak averaging, the intensity of connected pixels that pass the thresholding are replaced by their pixel averaged intensity. This is effective in revealing the order parameters when the intensity of the peaks are much larger than the intensity of the surrounding diffuse scattering. For the analysis of $Cd_2Re_2O_7$, the diffuse scattering intensities are orders of magnitude less than the peak centres [see Fig. 3(c)] and this justifies the peak averaging modality of *X-TEC*-s for that analysis.

F. Visualization of clustering results. After running the *X-TEC*-s or *X-TEC*-d algorithm, the next step is to visualize the results, through the cluster mean and variance, and the cluster assignments of the pixels in $\vec{q}$ space. Following this step, the user can interpret and eliminate the uninteresting clusters such as those features that correspond to detector artifacts, and identify those clusters that are physically interesting.

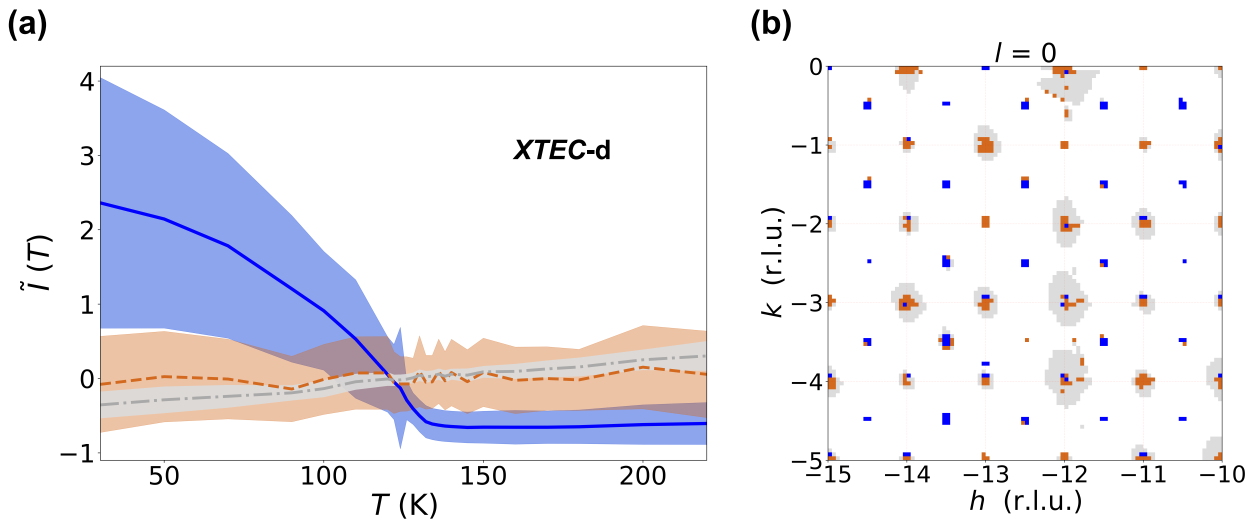

Fig. S4. *X-TEC*-d results of $Sr_3Rh_4Sn_{13}$ data with $K = 3$ clusters. **(a)** The cluster trajectories with clustering assignments color-coded as blue, brown and grey. $\tilde{I}(T)$ are the rescaled intensities by dividing each intensity trajectory with its mean over temperature and subtracting one. The lines represent cluster means and the shaded region shows one standard deviation. **(b)** The corresponding cluster assignments in the $(h, k, 0)$ plane, with pixels color coded according to their cluster assignments.

In Fig S4, we show the *X-TEC*-d results of the $Sr_3Rh_4Sn_{13}$ data using $K = 3$ clusters. The cluster assignments are labeled through different colors. The characteristic temperature trajectory of each cluster is given by the cluster mean [lines in Fig. S4(a)] and the uncertainty in the clustered trajectories are given by the cluster variance [shaded region in in Fig. S4(a) show one standard deviation]. Clusters whose means are well separated from others beyond their standard deviation are robust trajectories, as is the order parameter like (blue) cluster in Fig. S4.

The cluster assignments in $\vec{q}_i$ space are shown in Fig. S4(b) where the pixels are assigned the colors according to their cluster assignments. We find that without label smoothing, Fig S4(b) shows neighboring pixels within the peak are often assigned to different clusters (blue and brown clusters). For the *X-TEC*-s results in the main text [Fig. 2(c,d)], label smoothing is applied after excluding the grey clusters identified from *X-TEC*-d This step leads to sharper results as we have ensured that the label smoothing does not spread far over to the diffuse scattering. Comparing Fig. 2 and Fig S4, we see that label

smoothing automatically harmonizes the assignments in the vicinity of blue and brown pixels at the cost of weakening the cluster separation.

G. Determining optimal number of clusters. In order to determine the optimal number of clusters that efficiently reveal the distinct trajectories, one has to vary the number of clusters K to a point where further increase in K has no effect in uncovering qualitatively different trajectories. For the *X-TEC*-d analysis of $Sr_3Rh_4Sn_{13}$ data, as apparent in Fig. S5, $K = 3$ is the optimal number for resolving the order parameter trajectory robustly. With $K = 2$, the overlapping trajectories indicate that the clusters are not fully resolved. On the other hand $K = 4$ show more clusters resolved within the noisy trajectories, with nearly identical trajectories and overlapping variances indicative of over fitting.

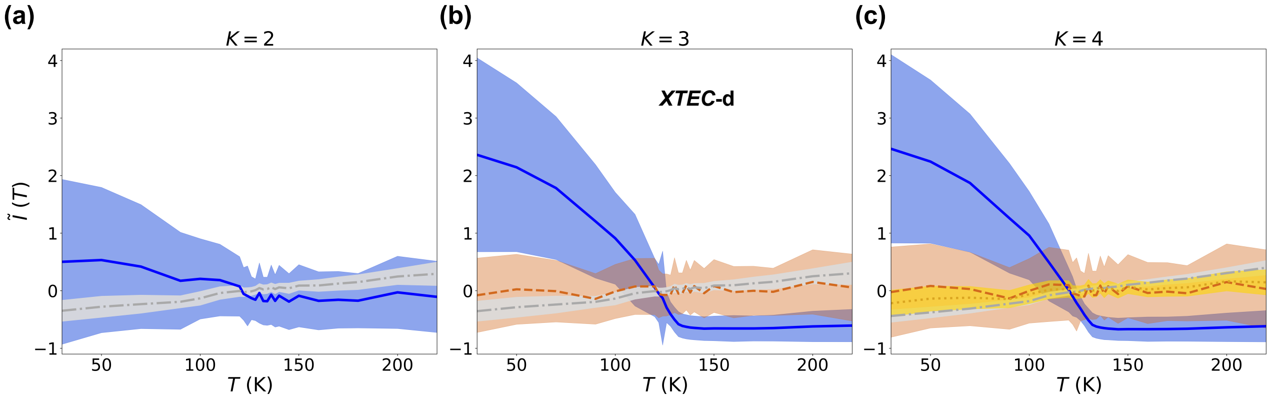

Fig. S5. *X-TEC*-d results on $Sr_3Rh_4Sn_{13}$ data, showing the mean trajectories (lines) and one standard deviation (shaded region) with **(a):** $K = 2$, **(b):** $K = 3$, **and (c):** $K = 4$. **clusters.**

H. Derivation of EM algorithm for GMM and general proof of convergence.. We follow derivations in Refs. 2 and 3. First recall Jenson's inequality: for convex function f and random variable X, $\mathbb{E}[f(X)] \geq f(\mathbb{E}[X])$ where for strictly convex functions, equality holds iff $X = \mathbb{E}[X]$ almost surely. Let $\ell(\theta)$, denote the model log-likelihood and X be our dataset with $x_i \in X$. Then

$$\begin{aligned} \ell(\theta) &= \log p(X;\theta) = \sum_i \log p(x_i;\theta) = \sum_i \log \sum_{z_i} p(x_i, z_i;\theta) \\ &= \sum_i \log \sum_{z_i} q_i(z_i) \frac{p(x_i, z_i;\theta)}{q_i(z_i)} \geq \sum_{i,z_i} q_i(z_i) \log \frac{p(x_i, z_i;\theta)}{q_i(z_i)} \equiv \tilde{\ell}(q,\theta) \end{aligned} \quad [5]$$

where $q_i(z_i)$ is some distribution over a random variable z_i (in our case this will be the cluster assignment) s.t. $\sum_{z_i} q_i(z_i) = 1$ and we have used Jenson's inequality. In order for this bound to be tight, $X = \mathbb{E}[X] \implies q_i(z_i) = p(z_i|x_i;\theta)$. Tightness of this bound implies that improving $\tilde{\ell}(q,\theta)$ necessarily improves $\ell(\theta)$ but since theta is unknown, we will have to make a guess, θ_t, and improve it iteratively. This iterative prescription is known as expectation maximization (EM). It consists of an E-step, where $q_i^t \leftarrow p(z_i|x_i;\theta_t)$ and an M-step $\theta^{t+1} \leftarrow \underset{\theta}{\text{argmax}}\, \tilde{\ell}(q^t,\theta)$.

We now derive the EM algorithm for the GMM. The E-step follows directly from the model likelihood and Bayes' theorem:

$$\begin{aligned} w_i^k &\equiv p(z_i = k|x_i;\pi_k,\mu_k,\Sigma_k) = \frac{\pi_k \mathcal{N}(x_i|\mu_k,\Sigma_k)}{\sum_k \pi_k \mathcal{N}(x_i|\mu_k,\Sigma_k)} \\ \mathcal{N}(x_i|\mu_k,\Sigma_k) &\equiv \frac{1}{(2\pi)^{n/2}} \frac{1}{\sqrt{\det \Sigma_k}} e^{-\frac{1}{2}(x_i-\mu_k)^\dagger \Sigma_k^{-1}(x_i-\mu_k)} \end{aligned} \quad [6]$$

For the M-step, we must find $\{\pi,\mu,\Sigma\}$ that optimizes our lower log-likelihood bound:

$$\tilde{\ell}(\{w_i^k,\pi_k,\mu_k,\Sigma_k\}) = \sum_{i,k} w_i^k \log\left[\frac{\pi_k \mathcal{N}(x_i|\mu_k,\Sigma_k)}{w_i^k}\right] + \lambda(1 - \sum_k \pi_k) \quad [7]$$

where λ is a Lagrange multiplier constraining the mixing weights to sum to unity.

Solving for the mixing weights:

$$
\begin{aligned}
&0 = \partial_{\pi_j}\tilde{\ell} = \sum_{i,k} w_i^k \frac{1}{\pi_k}\delta_{jk} - \lambda \sum_k \delta_{jk} \implies \lambda = \frac{1}{\pi_j}\sum_i w_i^j \\
&\lambda = \lambda \sum_k \pi_k = \sum_{i,k} w_i^k = \sum_i 1 \equiv m \\
&\quad \implies \pi_j = \frac{1}{m}\sum_i w_i^j
\end{aligned}
\tag{8}
$$

Solving for the mean:

$$
\begin{aligned}
&0 = \partial_{\mu_l}\tilde{\ell} = 2\sum_i w_i^l \Sigma_l^{-1}(x_i - \mu_l) \\
&\implies \mu_l = \frac{1}{\sum_i w_i^l}\sum_i w_i^l x_i
\end{aligned}
\tag{9}
$$

Solving for the covariance is a little trickier. First note the following matrix identities for symmetric invertible matrix A:

$$
\begin{aligned}
&\partial\big(\log(\det A)\big) = \mathrm{Tr}(A^{-1}\partial A) \\
&\partial A^{-1} = -A^{-1}(\partial A)A^{-1}
\end{aligned}
\tag{10}
$$

Now, when solving for the covariance we promote the covariance cluster index to an upper index so that the lower indices refer to the matrix elements:

$$
\begin{aligned}
0 &= \partial_{\Sigma^l_{mn}}\tilde{\ell} = \sum_{i,k} w_i^l \partial_{\Sigma^l_{mn}}\Big[\log\det\Sigma^k + (x_i-\mu_k)^\dagger(\Sigma^k)^{-1}(x_i-\mu_k)\Big] \\
&= \sum_{i,k} w_i^l\Big[\delta_{lk}\,\mathrm{Tr}\left\{(\Sigma^{k-1})_{rs}\delta_{sm}\delta_{tn}\right\} - \delta_{lk}\sum_{ps}(x_i-\mu_k)^\dagger_p\Big\{\sum_{qr}(\Sigma^{k-1})_{pq}\delta_{mq}\delta_{nr}(\Sigma^{k-1})_{rs}\Big\}(x_i-\mu_k)_s\Big] \\
&= \sum_i w_i^l\Big[\Sigma^{l-1}_{nm} - \sum_{p,s}(x-\mu_l)^\dagger_p\Sigma^{l-1}_{pm}\Sigma^{l-1}_{ns}(x-\mu_l)_s\Big] \\
&= \sum_i w_i^l\Big[\Sigma^{l-1} - \Sigma^{l-1}(x_i-\mu_l)(x_i-\mu_l)^\dagger\Sigma^{l-1}\Big] \\
0 &= \sum_i w_i^l\Big[\Sigma^l - (x_i-\mu_l)(x_i-\mu_l)^\dagger\Big] \\
&\implies \Sigma_l = \frac{1}{\sum_i w_i^l}\sum_i w_i^l(x_i-\mu_l)(x_i-\mu_l)^\dagger
\end{aligned}
\tag{11}
$$

Note that all quantities derived about have the same form as one would expect from standard regression but with each data point x_i having a cluster weight w_i^k.

2. *X-TEC*-d analysis of $TiSe_2$ CDW ordering

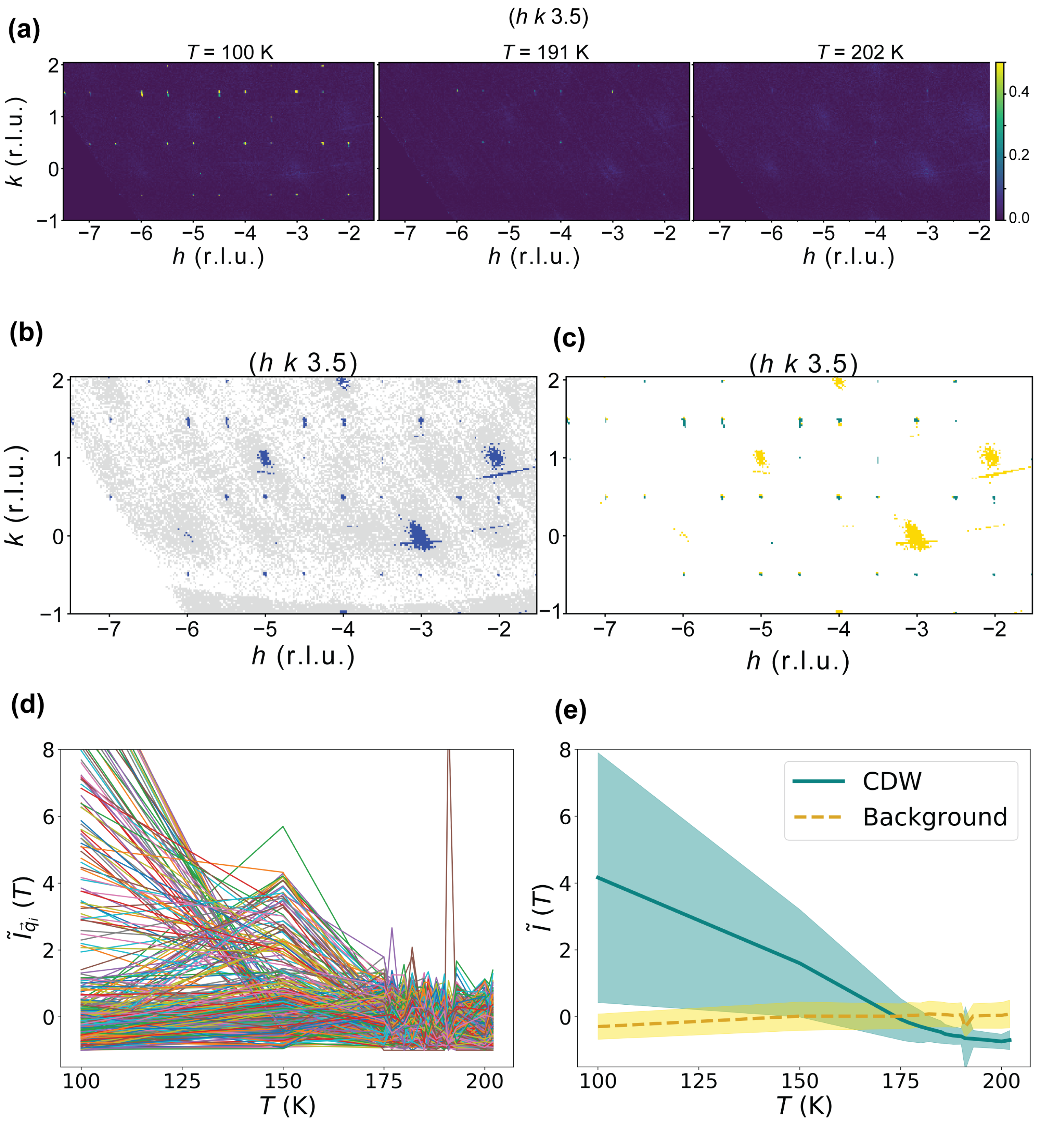

Fig. S6. *X-TEC*-d analysis of $TiSe_2$. $\mathbf{(a)}$ Two-dimensional slices of intensity of 1T-$TiSe_2$ on the $l = 3.5$ plane at three temperatures. This plane contains super-lattice peaks at $T < T_c = 200K$ (left) that disappears with the melting of the CDW order (right). (h, k, l) are in reciprocal lattice units (r.l.u.), and the color-map over saturates intensity (arb. unit.) > 0.5. $\mathbf{(b)}$ Thresholding described in SM. 1-B removes the grey clusters in the reciprocal space of the plane shown in (a). Only the blue clusters are tracked using *X-TEC*. $\mathbf{(c)}$ *X-TEC*-d two-cluster $(K = 2)$ results assign the colors yellow and teal to the blue pixels of (b). $K = 2$ is optimal to resolve all distinct trajectories. The locations of teal pixels identify with the CDW peaks, while yellow pixels identify with the background scattering. $\mathbf{(d)}$ Preprocessed intensity-temperature trajectories $\{\tilde{I}_{\vec{q}_i}(T)\}$ at $\{\vec{q}_i\}$s that passed the thresholding [blue pixels in panel (b)]. The intensities are re-scaled by their mean over T [SM. Eq. (2)]. The temperature T spans over $d^T = 14$ values, $\{T_1 = 100K, \cdots, T_{14} = 202K\}$. $\mathbf{(e)}$ From $\{\tilde{I}_{\vec{q}_i}(T)\}$, two distinct temperature trajectories (teal and yellow cluster) are resolved by *X-TEC*-d. Lines denote cluster means and shading represents cluster variance (one standard deviation) for the non-trivial CDW cluster (teal) and the background cluster (yellow), interpolated between the $d^T = 14$ temperatures measured.

This section provides another benchmarking example for X-TEC with a well-known CDW material: $TiSe_2$ (4, 5). Fig S6 shows the outcome of X-TEC-d applied to XRD data of 1T-$TiSe_2$, collected at the Cornell High Energy Synchrotron Source (CHESS). As a test case, we specifically explored intensities $I_{\vec{q}}(T_j)$ for $\{T_j\} \equiv \{T_1 = 100K, \cdots, T_{14} = 202K\}$ in the $(h, k, l = 3.5)$ plane. The raw XRD images in Fig. S6(a) show that superlattice peaks are present at $T = 100$K and disappears between $T = 191$K and 202K. The data undergoes a two-stage preprocessing: thresholding (SM section I-B) and rescaling. Thresholding removes low intensity noise and reduces the number of $\vec{q}$-space points to be canvassed from the full grid to a selection of points $\{\vec{q}_i\}$, containing meaningful features [see Fig. S6(b)]. Since the CDW peaks undergo a larger variation in temperature compared to the background as apparent from the raw images, the mean based rescaling [SM. Eq. (2)] is the right choice for this case. We now cluster the resulting collection of preprocessed temperature trajectories, $\tilde{\mathbf{I}}(\vec{q}_i) \equiv \{\tilde{I}_{\vec{q}_i}(T_j); j = 1, \cdots, d^T\}$ [see Fig. S6(d)], with X-TEC-d to discover qualitatively distinct types of temperature dependencies in the data. Two clusters are sufficient to meaningfully separate the distinct temperature trajectories. The clustered trajectories are represented by the cluster mean and variance, shown in Fig. S6(e). The contrast between the means of the teal cluster and the yellow cluster makes it evident that the teal cluster represents the order parameter and the temperature at which it crashes down is the critical temperature. The separation between the means exceeding the individual variance affirms the clustering to be a meaningful result. Interpretation of the X-TEC results is immediate upon locating the two clusters in reciprocal space, as shown in Fig S6(c), and inspecting the raw data. The locations of the yellow pixels identify the CDW wave vector to be $\vec{Q}_{CDW} = \{(0, \pi, \pi), (\pi, \pi, \pi))\}$, and equivalent momenta in the hexagonal basis. X-TEC thus detected the CDW transition with the correct transition temperature $T_c = 200$ K and correct ordering wavevector $\vec{Q}_{CDW}$(6) without any prior knowledge.

3. $Cd_2Re_2O_7$ Analysis

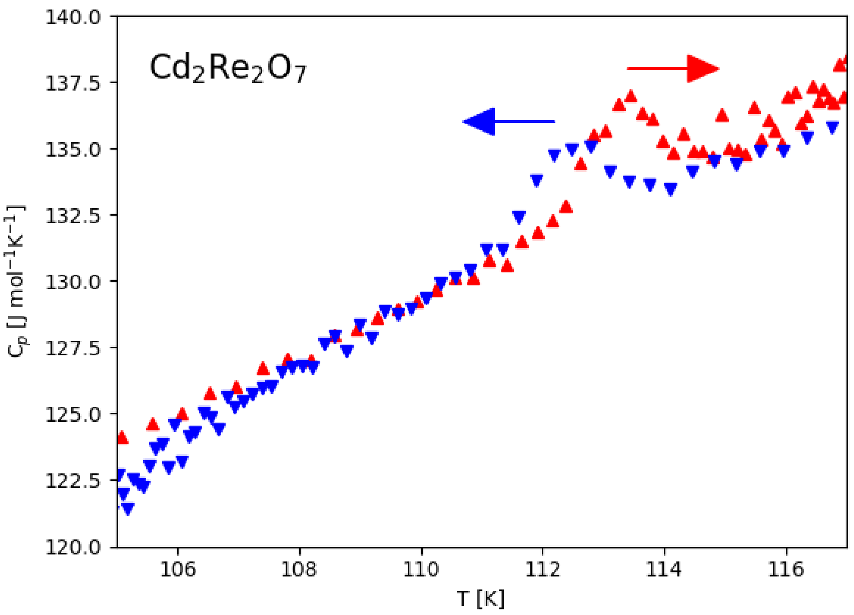

Fig. S7. Specific heat of $Cd_2Re_2O_7$, measured using the scanning method described in the text.

A. Specific Heat Measurements. In the main text, the heat capacity (C_p) of $Cd_2Re_2O_7$ was displayed in Fig. 3(b). The data shown in that figure was processed by a standard method in relaxation calorimetry ("pseudostatic method") in which the heat capacity is assumed to be constant throughout the heating and cooling segments of an applied heat pulse during which $\Delta T \lll$T. However, in the presence of a 1st order transition, the shape and magnitude of a peak in C_p at the phase transition temperature can be modified, while the hysteresis can be lost, when using the pseudostatic method. For this reason, we have also used the "scanning method" for which C_p is numerically determined at every point in the warming and cooling segments, which yields a more accurate peakshape and hysteresis for a 1st order transition at the cost of noise and absolute accuracy. A more detailed description of pseudostatic and scanning analysis can be found in Ref. 7. Fig. S7 shows the temperature dependence of C_p in the vicinity of the ~ 113 K phase transition when analyzed using the scanning method. A small but resolvable thermal hysteresis was observed between the peaks in C_p from the heating and cooling curves, which is suggestive of a latent heat and hence a first-order character. We do note, however, that the peak height and width of the peak in C_p did not differ substantially between these two methods, as would also be anticipated for a first-order transition, and for this reason the analysis of C_p alone is not definitive in identifying the order of the transition.

B. Low resolution $Cd_2Re_2O_7$ XRD data. This section discuss the *X-TEC* analysis with a lower resolution XRD data of $Cd_2Re_2O_7$. We first performed scans using an x-ray energy of 87 keV, which contained scattering spanning nearly 15,000 Brillouin zones, A first pass of *X-TEC*-s[*] for two clusters ($K = 2$) readily finds a cluster whose intensity rises sharply below $T_{s1} = 200$ K [the purple cluster in Fig. S8(a)]. The crisp clustering results with tight variance around the means reflect the amplification of the meaningful trend upon using data from a large number of BZ's. By examining the *X-TEC* cluster assignments, we find the purple cluster to exclusively consist of peaks with $\vec{Q} = (H, K, L)$, with all indices even, exactly one of which is not divisible by four, using the cubic indices of Phase I [see Fig. S8(b)]. Peaks that are equivalent in the cubic phase have different temperature dependence in Phase II, implying that the sample is untwinned, something that is confirmed by our high-resolution data. This means that the presence of $(00L)$ peaks with $L = 4n + 2$ below T_{s1} in phase II unambiguously rules out all the tetragonal space groups compatible with the pyrochlore structure, apart from $I\bar{4}m2$ and $I\bar{4}$. According to an earlier group theoretical analysis(8), of these two, only the former is compatible with a single second-order phase transition, so our data is strong confirmation of previous conclusions that, at T_{s1}, $I\bar{4}m2$ phase is selected out of two-dimensional E_u representation(9, 10).

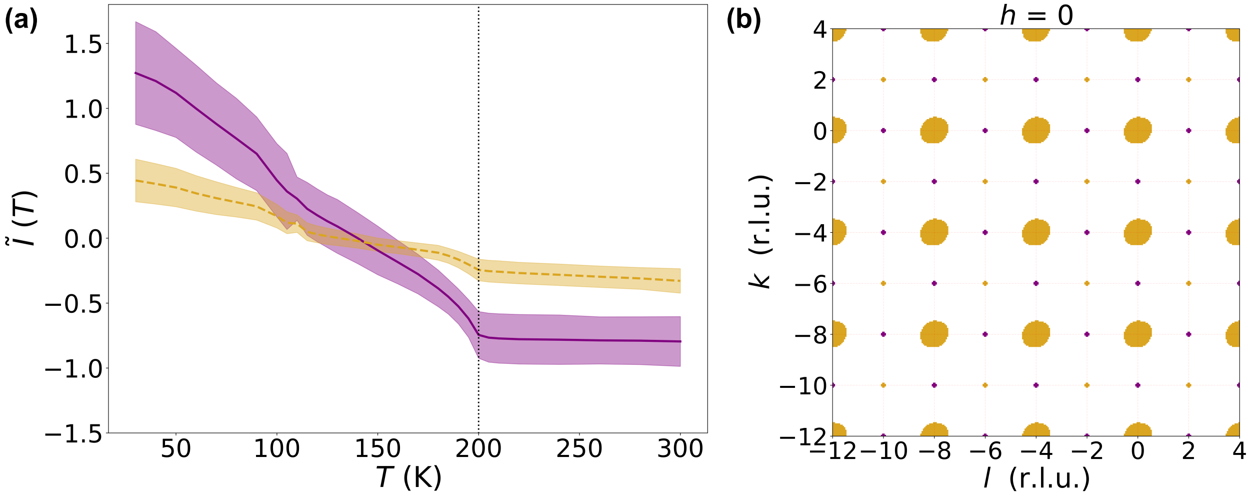

Fig. S8. Two-cluster *X-TEC*-s results on the lower resolution data spanning 15,000 BZ's of $Cd_2Re_2O_7$. **(a)** Cluster mean (lines) and one standard deviation (shaded areas) for the two clusters are shown in purple and yellow, interpolated between $d^T = 30$ temperature points of measurement. The data is peak averaged prior to the *X-TEC* preprocessing to suppress fluctuation signal and isolate the transition at T_{s2}. $\tilde{I}(T)$ denotes the mean rescaled intensity [Eq. (2)]. **(b)** The cluster assignements of thresholded $\vec{q}_i$ points that belong to the two clusters in (a) in a portion of the $h = 0$ plane.

C. Preprocessing and clustering setup details. Here we specify different preprocessing steps and clustering choices for the analysis of $Cd_2Re_2O_7$ high resolution data presented in Fig. 3 and 4 of main text, as well as the lower resolution data in Fig S8.

- ***X-TEC*-s (peak averaged) on cubic forbidden peaks of high resolution data: Fig 3(c), Fig 4(a)**

  1. We begin by selecting a $50 \times 50 \times 50$ region around each known peak center and thresholding as described in SM 1-B.
  2. We then floodfill from the peak centers and average all resulting trajectories to form a single, averaged trajectory per peak.
  3. We rescale the data by z-scoring it.
  4. We exclude all the cubic allowed Bragg peaks, and restrict the temperature range to [30 K, 150 K] so that *X-TEC* can focus on better resolving the distinct cluster trajectories across T_{s2}. See Fig S9 for the same analysis, but including all Bragg peaks and over the full temperature range.
  5. We cluster the peak-averaged trajectories using K = 2 clusters. We found two clusters to be the minimum number necessary to separate all distinct behaviors and that there was no advantage to using more than two.
  6. The dashed lines in Fig. 3(c) and symbols in Fig 4(a) show the cluster averaged intensity trajectory of the two clusters. The cluster averaged trajectory is shown for the full temperature range: [30 K, 300 K], although the clustering assignments were obtained from trajectories ≤ 150 K.

- ***X-TEC*-d (peaks opened) on cubic forbidden peaks of high resolution data: Fig 3(c-d), Fig: 4(c)**

  1. We select a $50 \times 50 \times 50$ window around each known peak center and threshold as described in SM 1-B.
  2. We only include peaks that are forbidden in the cubic phase. The temperature range is restricted to [30 K, 150 K] like in the peak averaged *X-TEC*-s analysis.

[*] here we simply averaged peaks due to the volume of the data.

3. We rescale the data by z-scoring it.
4. We cluster the data using $K = 3$ clusters.
5. The resulting cluster averaged intensity trajectory for the full temperature range: [30 K, 300 K] is shown as solid lines in Fig. 3(c).
6. Cluster averaging the absolute intensity trajectories washes out the characteristic temperature dependence of the diffuse halos. This can be remedied by cluster averaging over z-scored intensities. This is reported in Fig. 4(c).

- ***X-TEC*-s (peak averaged) on low resolution data: Fig S8**

1. In order to reduce noise, we first construct an average BZ mask by thresholding every BZ as described in SM 1-B and then averaging the thresholded BZs together.
2. We then manually select a cutoff value for the averaged BZ that maintains all the peaks while removing as much background as possible, and set each $\vec{q}$-point in the average BZ with value greater than the cutoff to 1, and the rest to 0 to form the mask.
3. We multiply each BZ by the average BZ mask to remove noise and emphasize the peaks.
4. Beginning from the known peak centers, we floodfill to pick out all $\vec{q}$-points belonging to each peak.
5. We perform peak averaging by averaging the trajectories of all $\vec{q}$-points belonging to each peak and replacing them with the single, averaged trajectory.
6. We apply the mean based rescaling [Eq. (2)] to the intensities.
7. Finally we cluster using K = 2 clusters. We subtract the minimum value of the cluster means when plotting to emphasize the order-parameter like behavior of the purple cluster in Fig. S8(a). Here *X-TEC* analyses the data for the full temperature range [30 K, 300 K].

- **Processing times for *X-TEC*** The *X-TEC*analysis of the higher resolution XRD data for $Cd_2Re_2O_7$ is the most time consuming of all the cases studied in this paper, and takes ~ 10 min in total to run.

1. The slowest step is to load the data. This step can be made faster by parallelization and eliminating regions in BZ that have no interesting physics. For our analysis, the relevant data is contained in the $50 \times 50 \times 50$ window around each Bragg peak, amounting to 17 GB of data. This takes $\sim 3000s$ to load with a single thread ($\sim 100s$ using 24 threads).
2. The GMM clustering takes $\sim 500s$ on a single thread with Intel(R) Xeon(R) CPU @ 2.60GHz.

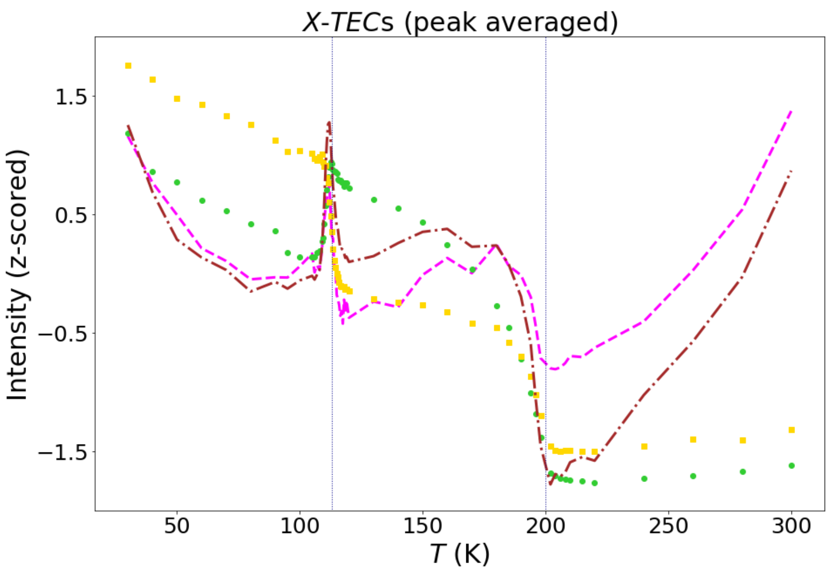

Fig. S9. Four-cluster *X-TEC*-s (peak averaged) results on the high resolution measurements of $Cd_2Re_2O_7$ retaining all Bragg peaks. Two of these sub-cluster trajectories (yellow and green symbols) identify with the cubic forbidden trajectories shown in Fig. 3(c) and Fig. 4(a) of main text. The other two sub-cluster trajectories (magenta and brown lines) arise from peaks that are not forbidden in the high-temperature cubic phase. The temperatures of the two structural phase transitions are shown as dotted lines.

D. Structure Factor Analysis. Fig 3 and 4 of main text discuss the two-clustering *X-TEC*-s analysis after excluding the cubic allowed peaks. By including all Bragg peaks, four clusters are sufficient for *X-TEC*-s to identify all the distinct trajectories. Fig. S9 shows the cluster means (z-scored intensity trajectories) for all four clusters identified by *X-TEC*-s (peak averaged) analysis. Two of these sub-clusters (yellow and green symbols) can be identified with the behavior of the cubic excluded peaks. It should be noted that these clusters represent the average temperature dependence of all the peaks assigned to their respective clusters, so there can be large variations within each cluster. However, the ML analysis has identified distinctive behavior in each cluster that we have verified by manual inspection of a number of peaks. All four clusters show similar temperature dependence close to the transition at $T_{s1} = 200$ K, but strikingly different behavior at the lower transition at $T_{s2} = 113$ K. The yellow cluster trajectory show a sudden drop while the green cluster peaks show a sudden increase in intensity across T_{s2}. The magenta and brown lines show a sharp spike in intensity at T_{s2}, before falling back to their values just above the transition. We do not currently have an explanation for this remarkable behavior.

The structural phase transition at T_{s1} is from the cubic pyrochlore structure, with space group $Fd\bar{3}m$, to a distorted tetragonal structure, with space group $I\bar{4}m2$. This space group allows distortions of the cadmium and rhenium cations along the z direction and either the x or y direction depending on the Wyckoff positions, using the $I\bar{4}m2$ unit cell, which is rotated by 45° from the cubic unit cell, *i.e.*, x is parallel to the (110) direction of the high-temperature cubic structure. There are associated displacements of the oxygen ions, but the x-ray measurements are not sensitive to them.

Analytic calculations of the structure factors for the Bragg peaks in terms of the allowed x and z distortions fall into four groups that correspond well to the four ML clusters. For example, the two groups whose intensities are forbidden in the high-temperature cubic phase (yellow and green) have the following form (H,K,L in following equations are in tetragonal indices):

$$F_1(H,K,L) \propto (-1)^{n_3} \sum_{M=\mathrm{Cd,Re}} \left\{ f_M \left[(-1)^{n_1} cos(2\pi H \delta x_M) e^{-2\pi i L \delta z_M} - (-1)^{n_2} cos(2\pi K \delta x_M) e^{2\pi i L \delta z_M} \right] \right\} \quad [12]$$

where $n_1 = \frac{1}{2}H$, $n_2 = \frac{1}{2}K$, and $n_3 = \frac{1}{4}(L-2)$.

$$F_2(H,K,L) \propto (-1)^{n_3} \sum_{M=\mathrm{Cd,Re}} \left\{ f_M \left[(-1)^{n_1} sin(2\pi H \delta x_M) e^{-2\pi i L \delta z_M} + (-1)^{n_2} sin(2\pi K \delta x_M) e^{2\pi i L \delta z_M} \right] \right\} \quad [13]$$

where $n_1 = \frac{1}{2}(H-1)$, $n_2 = \frac{1}{2}(K-1)$, and $n_3 = \frac{1}{4}L$.

It can be seen that, for small values of H and K, $F_1(H,K,L)$ are mostly sensitive to distortions along the z-axis, whereas for small values of L, $F_2(H,K,L)$ is mostly sensitive to in-plane distortions along x or y (where $\delta x = \delta y$). The assignments of individual peaks in the *X-TEC* analysis show that the (H,K,L) values of the green cluster are indeed dominated by in-plane distortions whereas the yellow cluster peaks are dominated by z-axis distortions. This suggests that the distinctive temperature dependences of peaks in the green and yellow clusters can be used to derive information about the relative distortions along x and z. If we assume that the temperature dependence of δx and δz follows that expected for an order parameter with a common critical exponent, β, from 200K down to 120K, the peak intensities would vary as $(T - T_c)^{2\beta}$.

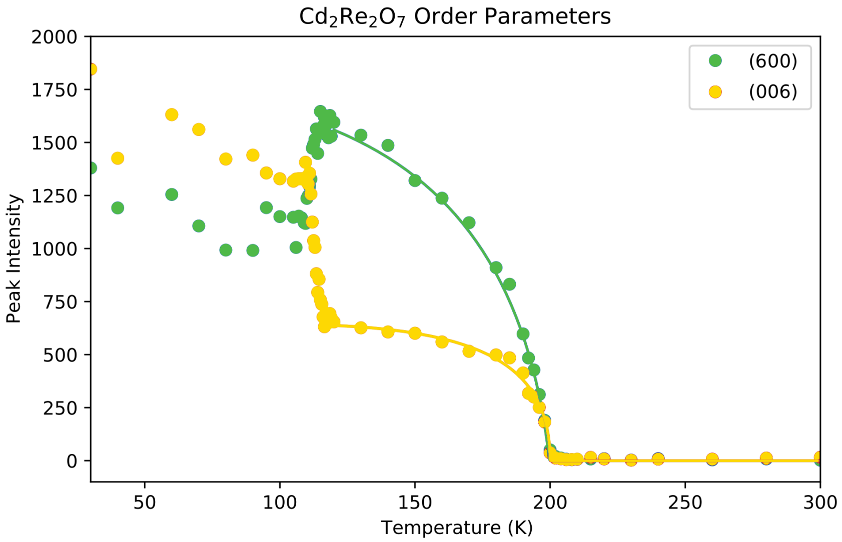

Fig. S10. Temperature dependence of the 006 (yellow) and 600 (green) Bragg peaks using cubic indices (006 and 3-30 using tetragonal indices). The green and yellow solid lines are fits between 120 K and 300 K to the structure factors in equations 10 and 11, respectively, assuming that the distortions, δx and δz for the Cd and Re ions, vary as $(T - T_c)^{\beta}$, with $\beta = 0.25$ and $T_c = 200$ K.

As an example, Figure S10 compares the 006 and 060 Bragg peaks using the indices of the cubic phase. These are the peaks that have been assigned to the yellow and green clusters of X-TEC-s shown in Fig. 4(a). Equations 10 and 11 show that the 006 (yellow) peak is only sensitive to δz_{Cd} and δz_{Re}, whereas the 060 (green) peak is only sensitive to in-plane distortions. The fit to the 006 peak yields relative z-axis distortions that are equal and opposite, *i.e*, $\delta z_{\mathrm{Re}} = -\delta z_{\mathrm{Cd}}$, illustrated in Fig. 4(b). The out-of-phase distortions are the reason for the flattening of the peak intensity of the 006 peak between 180 K and 120 K, confirming the conclusions based on the fits to the cluster means in Fig. 4(a). On the other hand, the 060 peak follows the scaling law from 200 K to 120 K, showing either that δx_{Re} has the same sign as δx_{Cd} or that one of the distortions is much larger than the other. This is an example where the temperature dependence of the peak intensities below a structural phase transition yields information on the relative internal distortions, which have proved to be too subtle for conventional crystallographic refinement until now.

E. Mode energies and intensities from Landau theory. The Landau free energy in an E_u model for $Cd_2Re_2O_7$ is (8)

$$F = a_1Q^2 + a_2Q^4 + a_3Q^6 + a_4Q^8 + Q^6[b_1 + cQ^2]\frac{1}{2}[1 + \cos(6\phi)] \quad [14]$$

with Q the order parameter amplitude and ϕ the phase angle. For $I\bar{4}m2$, ϕ = 30(2n+1) and for $I4_122$, ϕ = 60n (the different angles represent different domains). F vanishes at T_{s1} and the anisotropy (last term) would vanish at T_{s2} if it were not for the first-order jump in Q. The Goldstone (phase) mode energy is given by (11)

$$\omega_G^2 = \chi_G^{-1}/M \quad [15]$$

where the inverse Goldstone susceptibility is

$$\chi_G^{-1} = \frac{1}{Q^2}\frac{\partial^2 F}{\partial \phi^2} = 18Q^4|b_1 + cQ^2| \quad [16]$$

with M some ion effective mass, where in the second expression we have taken into account the value of ϕ in the two phases (which leads to the modulus). The Higgs mode energy is given by

$$\omega_H^2 = \chi_H^{-1}/M \quad [17]$$

where

$$\chi_H^{-1} = \frac{\partial^2 F}{\partial Q^2} = 2a_1 + 12a_2Q^2 + 30a_3Q^4 + 56a_4Q^6 + 30Q^4[b_1 + \frac{28}{15}cQ^2]\frac{1}{2}[1 + \cos(6\phi)] \quad [18]$$

The first-order transition from $I\bar{4}m2$ (phase II) to $I4_122$ (phase III) is given by the condition (8)

$$a_1 = 2a_2(b_1/c) - 3a_3(b_1/c)^2 + (4a_4 + c/2)(b_1/c)^3 \quad [19]$$

Q^2 is given by the cubic equation ($\frac{\partial F}{\partial Q} = 0$)

$$-2a_1 = 4a_2Q^2 + 6a_3Q^4 + 8a_4Q^6 + 6Q^4[b_1 + \frac{4}{3}cQ^2]\frac{1}{2}[1 + \cos(6\phi)] \quad [20]$$

Finally, the soft mode energy above T_{s1} (ω_s) is gotten by setting Q=0 in χ_H^{-1}. In practice, the effective M is unknown (involving Cd, Nb and O masses), so all mode energies will be multiplied by the same constant in order to agree with Raman data (12) for the Higgs energy at T=0 (85 cm^{-1}).

We now have all we need to calculate the order parameter, the phase boundary, and mode energies. What about the mode intensities? The basic idea can be seen from the work of Fleury (13) and Shapiro (14). The energy integrated intensity (appropriate for the diffuse scattering collected from high energy x-rays) is given by (14)

$$I_q = \frac{1}{\pi}\int [n(\omega) + 1]\Im[\omega_q^2 - \omega^2 - i\omega\Gamma_q]^{-1}d\omega \quad [21]$$

where $n(\omega)$ is the Bose factor, ω_q is the mode energy for a given q, and Γ_q is the lifetime broadening. Assuming we can replace $n(\omega)$ by T/ω, this integral reduces to

$$I_q = T/\omega_q^2 \quad [22]$$

This expression is obviously divergent for q=0 at T_{s1}. To correct for this, we recognize that the data are collected over a small range in q. We assume the q dependence of the mode energy goes like

$$\omega_q^2 = \omega_0^2 + \alpha^2q^2 \quad [23]$$

where α results from the gradient terms in the Landau energy. Integrating over q, we obtain

$$T\int \frac{q^2dq}{\omega_q^2} \propto T[1 - \tilde{\omega}_0 \tan^{-1}\frac{1}{\tilde{\omega}_0}] \quad [24]$$

where $\tilde{\omega}_0 = \frac{\omega_0}{\alpha q_c}$ with q_c the momentum cut-off. Since α is unknown (no mode dispersions have been measured for this material), we set αq_c to the lower bound of the Raman data (6 cm^{-1} (12)) for all modes.

Now for the matrix elements. That is, how do the x-rays couple to the modes? We assume unit coupling to the Higgs and soft modes, the Higgs mode below T_{s1} being the analog of the soft mode above T_{s1}. But for the Goldstone mode, which only exists below T_{s1}, we set the coupling constant to Q^2 (13). So, above T_{s1} we have

$$T[1 - \tilde{\omega}_s \tan^{-1} \frac{1}{\tilde{\omega}_s}] \quad [25]$$

and below T_{s1} we have

$$T[1 - \tilde{\omega}_H \tan^{-1} \frac{1}{\tilde{\omega}_H} + Q^2(1 - \tilde{\omega}_G \tan^{-1} \frac{1}{\tilde{\omega}_G})] \quad [26]$$

To evaluate, we choose parameters as in Fig. 3b of (8), with b_1=0.3. We then do the following normalizations. a_1 is some constant times $T - T_{s1}$. This constant is adjusted so that T_{s1} is 200 K and T_{s2} is 113 K. Then the mode energies are normalized as stated above (so that the Higgs mode energy is equal to 85 cm^{-1} at T=0 as observed by Raman (12)). Finally, the intensities are normalized by T_{s1}. In Fig. S11, the resulting mode energies and intensities are shown. Note the small jump in the Higgs energy and the dip in the Goldstone energy at T_{s2}. Also that the Goldstone intensity completely dominates outside the critical region associated with T_{s1}. As an aside, the Raman data cut-off at about 6 cm^{-1} as noted above. The prediction is that the Goldstone mode energy should rise above this value at low T. We suggest then that the Raman mode seen at 30 cm^{-1} below T_{s2} could be the Goldstone mode. This in turn implies that the central peak in the intensity from Raman has more contributions to it than the Goldstone one, and this would presumably be due to elastic scattering from impurities and static short-range structural disorder.

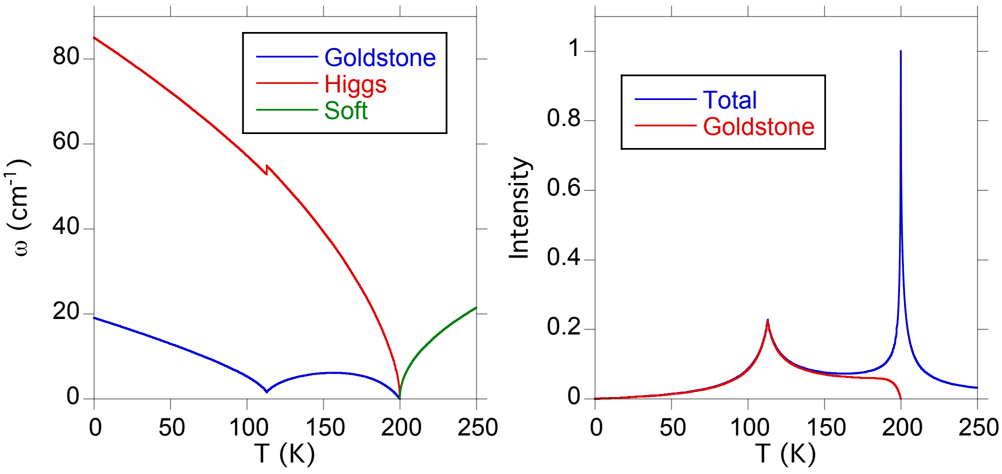

Fig. S11. (a) Landau mode energies as a function of T for $Cd_2Re_2O_7$. Note the first order jump in the Higgs energy and the dip in the Goldstone energy at T_{s2} (113 K). (b) Landau mode intensities as a function of T. Outside of the critical region near T_{s1} (200 K), the intensity is dominated by the Goldstone intensity. Note the resemblance of the calculated intensity to the XRD diffuse scattering intensity presented in this paper (Fig. 4(c)).

Finally, some caveats. First, the behavior well below T_{s2} cannot be taken too seriously since Landau theory is not valid at low T where $Q(T)$ flattens as a function of T (as observed for the Higgs mode by Raman). Nor for the intensities where the T/ω approximation for $n(\omega)$ is not valid. Second, the theory is for a pure E_u model. In reality, the secondary mode A_{2u} (corresponding to distortions along the $< 111 >$ trigonal axis orthogonal to E_u distortions) will play some role, and its coupling to E_u is also an anisotropy term in the Landau energy (it does not exist for $I4_122$) (8). Finally, the critical exponent near T_{s1} is the mean field one. In reality, experiment finds β=1/4, not 1/2. Despite these caveats, Fig. S11 is remarkably similar to the Raman data, and the XRD data reported in this paper. This brings into question the interpretation of the pump-probe measurements in Ref. (15) which claims that a structural soft mode does not exist for $Cd_2Re_2O_7$.

References

1. Krogstad MJ, et al. (2020) Reciprocal Space Imaging of Ionic Correlations in Intercalation Compounds. *Nature Materials* 19(1):63–68.
2. Murphy KP (2013) *Machine learning : a probabilistic perspective.* (MIT Press, Cambridge, Mass. [u.a.]).
3. Ng A (2017) Cs229 lecture notes. *CS229 Lecture notes.*
4. Salvo FJD, Moncton DE, Waszczak JV (1976) Electronic properties and superlattice formation in the semimetal TiSe$_2$. *Phys. Rev. B* 14:4321.
5. Wilson JA, Yoffe AD (1969) The transition metal dichalcogenides discussion and interpretation of the observed optical, electrical and structural properties. *Adv. Phys.* 18(73):193 – 335.

6. Di Salvo FJ, Moncton DE, Waszczak JV (1976) Electronic properties and superlattice formation in the semimetal $tise_2$. *Phys. Rev. B* 14(10):4321–4328.
7. Gillard TM, Phelan D, Leighton C, Bates FS (2015) Determination of the Lamellae-to-Disorder Heat of Transition in a Short Diblock Copolymer by Relaxation Calorimetry. *Macromolecules* 48(13):4733–4741.
8. Sergienko IA, Curnoe SH (2003) Structural Order Parameter in the Pyrochlore Superconductor $Cd_2Re_2O_7$. *J. Phys. Soc. Jpn* 72(7):1607 – 1610.
9. Petersen JC, et al. (2006) Nonlinear optical signatures of the tensor order in $Cd_2Re_2O_7$. *Nature Physics* 2(9):605–608.
10. Yamaura Ji, et al. (2017) Successive spatial symmetry breaking under high pressure in the spin-orbit-coupled metal $Cd_2Re_2O_7$. *Phys. Rev. B* 95(2):020102.
11. Meier QN, et al. (2020) Manifestation of structural higgs and goldstone modes in the hexagonal manganites. *Phys. Rev. B* 102(1):014102.
12. Kendziora CA, et al. (2005) Goldstone-mode phonon dynamics in the pyrochlore $Cd_2Re_2O_7$. *Phys. Rev. Lett.* 95(12):125503.
13. Fleury PA (1976) The effects of soft modes on the structure and properties of materials. *Annual Review of Materials Science* 6(1):157–180.
14. Shapiro SM, Axe JD, Shirane G, Riste T (1972) Critical neutron scattering in $srtio_3$ and $kmnf_3$. *Phys. Rev. B* 6(11):4332–4341.
15. Harter JW, et al. (2018) Evidence of an improper displacive phase transition in $Cd_2Re_2O_7$ via time-resolved coherent phonon spectroscopy. *Phys. Rev. Lett.* 120(4):047601.
